# Machine Learning and Deep Learning for the Diagnosis of Cervical Degenerative Diseases: Systematic Review and Meta-Analysis

**DOI:** 10.2196/92008

**Published:** 2026-10-05

**Authors:** Hongwei Duan, Ruiyuan Chen, Minghui Liang, Liqian Wang, Tianyi Wang, Aobo Wang, Ziqian Ma, Yu Xi, Shuo Yuan, Ning Fan, Lei Zang

**Affiliations:** 1 Department of Orthopedics, Beijing Chao-Yang Hospital, Capital Medical University, 5 JingYuan Road, Shijingshan District, Beijing, China, 86 51718688

**Keywords:** cervical degenerative diseases, artificial intelligence, deep learning, machine learning, traditional machine learning, diagnosis

## Abstract

**Background:**

Cervical degenerative diseases are a global public health issue, and their incidence is rising worldwide. Although an increasing number of studies on traditional machine learning (TML) and deep learning (DL) have been conducted in the detection and segmentation of cervical degenerative diseases and have reported promising task-specific results, the performance of these models has not yet been systematically analyzed.

**Objective:**

This systematic review and meta-analysis aimed to summarize and evaluate existing evidence on TML and DL approaches for diagnosing cervical degenerative diseases, thereby comprehensively guiding future research and clinical applications.

**Methods:**

This systematic review was conducted in accordance with the PRISMA (Preferred Reporting Items for Systematic Reviews and Meta-Analyses) guidelines. A comprehensive literature search was conducted on PubMed, Embase, the Cochrane Library, Web of Science, Scopus, and the Institute of Electrical and Electronics Engineers (IEEE Xplore) from January 2000 to June 2026, supplemented by backward and forward citation searching in Scopus. Studies evaluating TML and DL algorithms for diagnosing cervical degenerative diseases using medical imaging were included. Methodological quality was assessed using the Quality Assessment of Diagnostic Accuracy Studies 2 (QUADAS-2) tool and the Quality Assessment of Diagnostic Accuracy Studies AI (QUADAS-AI) tool. For the primary diagnostic accuracy meta-analysis, data were synthesized using a bivariate mixed-effects logistic regression model. Sensitivity and specificity were summarized separately using random-effects meta-analysis with the Knapp-Hartung adjustment, and 95% prediction intervals (PIs) were reported. Certainty of evidence was assessed using the GRADE (Grading of Recommendations Assessment, Development and Evaluation) approach.

**Results:**

This systematic review included 30 studies, of which 21 involved a total of 25,301 patients included in the meta-analysis. The pooled sensitivity and specificity were 0.92 (95% CI 0.89‐0.96; 95% PI 0.80‐1.00) and 0.88 (95% CI 0.84‐0.91; 95% PI 0.72‐1.00), respectively. The positive likelihood ratio (LR) was 8.36 (95% CI 6.14‐11.36), and the negative LR was 0.07 (95% CI 0.04‐0.11). The area under the summary receiver operating characteristic (SROC) curve was 0.96 (95% CI 0.94‐0.97). Leave-one-out analyses did not materially alter the pooled estimates. High risk of bias was identified in 4 studies using QUADAS-2 and in 17 using QUADAS-AI. The overall certainty of evidence was rated as low according to the GRADE approach.

**Conclusions:**

TML and DL models demonstrated satisfactory diagnostic performance for cervical degenerative diseases, although external validation was limited. Unlike previously published reviews in this field, this study provides pooled estimates of the diagnostic performance of TML and DL for cervical degenerative diseases and indicates that, given between-study heterogeneity and low certainty of evidence, AI should currently be used as clinical decision support rather than an independent replacement for physicians.

## Introduction

Cervical degenerative diseases mainly encompass degenerative cervical spondylosis, primarily characterized by disc herniation, osteophyte formation, and ligamentous hypertrophy [1]. These structural alterations form the anatomical basis for the subsequent involvement of adjacent neurovascular structures [1]. Cervical degenerative diseases have become a global public health issue, as their incidence and prevalence continue to increase worldwide, with a progressively earlier age of onset [2,3]. Common symptoms and signs include varying degrees of soreness and pain in the neck and shoulder region, radiating numbness in the upper limbs, lower-limb weakness with a “stepping-on-cotton” sensation, as well as headache, dizziness, palpitations, and blurred vision [4]. Accurate identification and assessment of cervical degenerative diseases are crucial for understanding disease progression, developing intervention strategies, and evaluating therapeutic outcomes. Clinical diagnosis typically relies on a comprehensive assessment of the patient’s medical history, physical examination results, and imaging studies, including radiography, computed tomography (CT), and magnetic resonance imaging (MRI) [5]. As objective evidence, imaging plays an irreplaceable and pivotal role in the detection and characterization of the presence, type, and severity of cervical degenerative diseases. However, manual interpretation of the large amount of detailed information contained in imaging studies is time-consuming and repetitive. In this context, more efficient, objective, and precise auxiliary tools are urgently needed to enhance the assessment of imaging features related to cervical degenerative diseases.

AI has been widely used for disease detection, segmentation, and classification tasks. Machine learning (ML) is a branch of AI [6]. Traditional ML (TML) methods rely on manually designed and extracted imaging features, including morphological features, texture features, and grayscale histograms. These features are classified using algorithms such as support vector machines, random forests, or logistic regression to identify and categorize imaging abnormalities [7]. Recently, deep learning (DL), a key branch of ML, has rapidly emerged. It leverages multiple processing layers to automatically learn complex imaging features directly from raw images, thereby reducing bias from manual intervention [8].

In 2019, Hopkins et al [9] initially attempted to apply deep learning to classify normal vs degenerative cervical spine disease on imaging. Subsequently, an increasing number of TML and DL studies have been conducted in the detection, segmentation, and classification of cervical degenerative diseases, achieving notable success [10-38]. Systematic reviews followed as this literature grew. Goedmakers et al [39] summarized the use of ML in cervical spine image analysis, with an emphasis on image segmentation and morphometric analysis. Stephens et al [40] reviewed broader applications of ML in cervical and lumbar degenerative diseases, including image analysis, patient selection, and postoperative outcome prediction. Vattipally et al [41] primarily examined clinical and functional data to review the use of ML in screening, clinical decision making, and prognosis for degenerative cervical myelopathy. Later reviews focused more directly on imaging diagnosis. Du et al [42] evaluated MRI-based AI models for degenerative cervical diseases, while Mougios et al [43] examined the performance of DL models for diagnosing cervical central spinal stenosis on MRI. In addition, 2 relevant quantitative reviews have been published. Wang et al [44] evaluated the performance of TML and DL for diagnosing lumbar spinal stenosis across multiple imaging modalities. Gete et al [45] evaluated the diagnostic accuracy of MRI-based DL models for degenerative diseases across spinal regions but included the cervical studies in the subgroup for other or mixed spinal regions.

Existing reviews have not provided a comprehensive quantitative synthesis focused specifically on the diagnostic performance of TML and DL for cervical degenerative diseases across MRI, radiography, and CT [39-45]. With the growing number of cervical imaging studies, sufficient evidence is now available to support a quantitative synthesis focused on cervical degenerative diseases. Most studies are retrospective and report model development or internal validation at a single center. Study populations, disease definitions, imaging modalities, model architectures, and validation strategies differ considerably. Reports are also often incomplete with respect to diagnostic accuracy, external validation, and reliability. Accordingly, strong performance in individual studies may not be reproduced in routine clinical practice. We conducted this systematic review and meta-analysis to evaluate the performance of TML and DL for diagnosing cervical degenerative diseases across MRI, radiography, and CT, quantify heterogeneity across studies, assess risk of bias and certainty of evidence, and clarify the potential role of these models in clinical decision support.

This systematic review and meta-analysis aimed to summarize and evaluate existing evidence on TML and DL approaches for the diagnosis of cervical degenerative diseases, thereby comprehensively guiding future research and clinical applications.

## Methods

### Protocol and Registration

This systematic review was conducted and reported in accordance with the PRISMA (Preferred Reporting Items for Systematic Reviews and Meta-Analyses) guidelines [46-48] and the PRISMA-DTA (Preferred Reporting Items for a Systematic Review and Meta-analysis of Diagnostic Test Accuracy Studies) statement [49]. The PRISMA 2020 for Abstracts, PRISMA 2020, and PRISMA-S (Preferred Reporting Items for Systematic Reviews and Meta-Analyses literature search extension) checklists are provided in Checklists 1–3, respectively. Its protocol was registered in PROSPERO (International Prospective Register of Systematic Reviews; ID: CRD420251266410). We made no amendments to the registered protocol. Ethical approval was not required owing to the retrospective nature of this review.

### Search Strategy

Records from 6 major databases, namely, PubMed, Embase, the Cochrane Library (CENTRAL), Web of Science Core Collection, Scopus, and IEEE Xplore, were collected from January 2000 to June 2026. The inclusion of IEEE Xplore was intended to improve coverage of AI-based diagnostic studies indexed outside biomedical databases. Each database was searched individually via its native interface; no databases were searched simultaneously on a single platform. The searches combined terms for the target condition with terms for the index test, consistent with Cochrane guidance for diagnostic test accuracy review [50]. Terms for the population, reference standard, comparator, and diagnostic outcomes were not mandatory because these concepts are inconsistently indexed or reported and could reduce search sensitivity.

Specifically, disease-related terms included “Cervical Spondylosis,” “Degenerative Cervical Myelopathy,” “Cervical Spinal Stenosis,” “Cervical Canal Stenosis,” “Cervical Foraminal Stenosis,” “Cervical Cord Compression,” “Cervical Disc Degeneration,” “Cervical Disc Herniation,” “Cervical Radiculopathy,” and related cervical degenerative disease terms. Artificial intelligence-related terms included “Artificial Intelligence,” “Machine Learning,” “Deep Learning,” “Neural Networks,” “Convolutional Neural Networks,” “Computer Vision,” “Computer-Assisted Diagnosis,” “Computer-Assisted Detection,” “Image Analysis,” “Pattern Recognition,” “Radiomics,” “Texture Analysis,” and related AI imaging terms. Details of the search strategy are provided in Multimedia Appendix 1. The search strategy was developed by HD and reviewed by LZ. No formal external peer review of the search strategy was sought.

In addition to the database searches, one round of backward and forward citation searching was conducted in Scopus after full-text eligibility assessment. All studies meeting the eligibility criteria and indexed in Scopus were used as seed reports. Backward citation searching was performed by screening the cited references of each seed report, whereas forward citation searching was performed by screening records that cited the seed reports in Scopus. Citation records were deduplicated within the citation set and against the database-search records. Two reviewers (HD and RC) independently screened the remaining records using the same eligibility criteria and publication date range applied to the database-search records. Citation searching was last conducted on June 23, 2026.

We did not search any study registries. We did not purposefully browse any online or print sources beyond the database searches and citation searching, and we did not use any additional information sources or search methods. The search strategies were developed specifically for this review and were not adapted from any previous reviews. We did not apply any published search filters. To maximize sensitivity, we did not restrict the search by language or study design; the only limit applied was the publication date range (January 2000 to June 2026). Non–English language publications were excluded during the screening phase, as stated in the eligibility criteria.

### Eligibility Criteria

The primary diagnostic question was framed using the participants, index test, and target condition (PIT) framework. The population was adults undergoing cervical spine imaging with suspected or confirmed cervical degenerative diseases. The index test was image-based TML or DL diagnostic models. The target condition was cervical degenerative diseases. In the context of image-based diagnosis, this umbrella term referred to degenerative structural changes assessable on cervical spine imaging, including cervical spinal canal stenosis, cervical neural foraminal stenosis, and cervical disc herniation. These imaging targets are cervical structural changes associated with degenerative processes, can be assessed and annotated on routine imaging such as MRI, CT, or X-ray, and served as the imaging basis for identifying, localizing, and grading cervical degenerative pathology in the included studies. Some original studies used disease level terms, such as degenerative cervical spondylosis, cervical spondylotic myelopathy, cervical spondylotic radiculopathy, and cervical degenerative disc disease. These terms were also considered under the umbrella term cervical degenerative diseases when their TML or DL models based on imaging ultimately evaluated the above radiographically visible degenerative changes. Eligible reference standards included expert image interpretation, established imaging criteria, operative or clinical diagnosis, and reference labels provided by the source dataset.

This review included studies evaluating the diagnostic performance of TML or DL in identifying cervical degenerative diseases using human imaging data, such as MRI, CT, or X-ray. These studies were required to directly provide a confusion matrix or be capable of reconstructing the confusion matrix. Studies that involved animal or phantom experiments or conducted only image processing without diagnosing cervical degenerative diseases were excluded. Non-English publications, studies lacking full text, gray literature, preprints, and studies with incomplete or duplicate data were also excluded. Furthermore, conference abstracts without sufficient data, study protocols, case reports, editorials, commentaries, and review papers, including systematic reviews and meta-analyses, were excluded.

### Selection Process

Two reviewers (HD and RC) independently searched and screened the literature. For reference management, all the retrieved records related to TML or DL models for diagnosing cervical degenerative diseases were imported into Zotero (Corporation for Digital Scholarship), and duplicates were removed. Then, titles and abstracts were screened to exclude clearly irrelevant studies. Subsequently, full texts of potentially eligible studies were carefully reviewed based on the predefined inclusion and exclusion criteria. Records identified through backward and forward citation searching were deduplicated against the database-search records and independently screened by the same two reviewers (HD and RC) based on titles and abstracts. Potentially eligible records were then assessed in full text using the predefined eligibility criteria. Any discrepancies were resolved through discussion or consultation with a third reviewer (LZ) when necessary. Studies were considered eligible for meta-analysis if they provided or allowed reconstruction of a confusion matrix. For studies included in the systematic review but lacking the required data for meta-analysis, the corresponding authors were contacted via email to obtain the necessary information.

### Data Collection

Two reviewers (HD and RC) independently extracted data using a standardized form, summarizing and organizing the following information: basic study characteristics, including publication year, study type, study design, model type, primary algorithms, and harmonized diagnostic categories based on the target conditions or imaging tasks reported in the original studies, data collection period, institution, sample size, study population, population demographics; population source; dataset split details; validation method; imaging modality; and diagnostic criteria. To avoid double counting of patient data, we explicitly traced and compared recruitment periods and research centers and study populations across studies. If various research used data from the same patient cohort, we prioritized the research with the most comprehensive follow-up or largest sample size, and excluded overlapping datasets from the analysis. All discrepancies were resolved by either discussion or, when necessary, consultation with a third reviewer (LZ). For studies reporting contingency tables for different types of cervical degenerative diseases, the contingency tables were assumed to be independent if the populations were nonoverlapping. For studies reporting contingency tables based on both internal and external validation datasets, the results from external validation were preferentially used. For the validation-strategy subgroup analysis, datasets obtained from an institution or independent data source different from that used for model development were classified as external validation. Random or temporal splits performed within the same institution were classified as internal validation. For studies with external test datasets, we compared the reported institution or data source, acquisition period, and patient population to determine whether the independence of external test data from training data could be verified and whether data leakage could be excluded. When these details were insufficiently reported, dataset independence was considered unclear. For studies reporting multiple contingency tables using different classifier algorithms or based on different preprocessing strategies, the best-performing result was used. The primary outcomes were diagnostic accuracy measures derived from confusion matrices, including sensitivity, specificity, and area under the receiver operating characteristic curve.

### Study Risk-of-Bias Assessment

All studies included in the meta-analysis were assessed for risk of bias. Two reviewers (HD and RC) independently evaluated the risk of bias across all domains using the Quality Assessment of Diagnostic Accuracy Studies 2 (QUADAS-2) and QUADAS-AI [51,52]. Both reviewers evaluated study quality using the predefined assessment tool, and any disagreements were resolved through discussion and consultation with a third reviewer (LZ).

### Synthesis Methods

Statistical analyses were conducted using Stata (version 19.5, StataNow/MP; StataCorp LLC) with its Meta-Analytical Integration of Diagnostic Accuracy Studies (MIDAS) module. For the primary diagnostic test accuracy meta-analysis, a bivariate mixed-effects logistic regression model was used to obtain pooled estimates of sensitivity, specificity, and diagnostic odds ratio, and to generate the summary receiver operating characteristic (SROC) curve for overall diagnostic accuracy. Given the anticipated clinical and methodological variability across the included studies, pooled sensitivity and specificity were separately summarized using random-effects meta-analysis with the Knapp-Hartung adjustment, and forest plots were constructed accordingly. Between-study variability was assessed using *I*^2^, while 95% prediction intervals (PIs) were calculated to describe the expected range of diagnostic performance across comparable settings. A leave-one-out sensitivity analysis was performed by iteratively removing one study at a time and recalculating the pooled sensitivity and specificity using the same random-effects model to evaluate the influence of individual studies. A Fagan nomogram and posttest probability curves were used to evaluate the association between pretest probability, likelihood ratios (LR), and posttest probability. The LR dot plots positioned each dataset according to established evidence thresholds for disease confirmation and exclusion. Subgroup analyses were conducted to examine the effects of validation strategy and imaging modality, with a minimum of 4 datasets required for each subgroup. Subsequently, a bivariate boxplot was used to examine the joint distribution of sensitivity and specificity across datasets, enabling further exploration of heterogeneity. Potential publication bias was assessed using Deeks funnel plot asymmetry test.

### Certainty Assessment

The certainty of evidence was evaluated using the GRADE (Grading of Recommendations Assessment, Development and Evaluation) methodology for diagnostic test accuracy studies, with downgrading for risk of bias, inconsistency, indirectness, imprecision, and publication bias [53].

## Results

### Study Selection and Characteristics

A total of 5085 records were identified through searches of 6 databases: PubMed (n=522), the Cochrane Library (n=29), Embase (n=3366), Web of Science (n=269), Scopus (n=844), and IEEE Xplore (n=55). After removal of 1051 duplicate records, 4034 records were screened, of which 3858 were excluded during title and abstract screening. Subsequently, 176 full-text articles were assessed for eligibility, and 146 articles were excluded for the following reasons: not traditional machine learning (TML) or deep learning (DL) studies (n=5), not based on medical imaging (n=63), not diagnostic studies (n=50), and not related to cervical degenerative diseases (n=28). None of the full-text articles were excluded on the basis of gray literature or preprint status. Of the 30 studies [9-38] included in the systematic review, 28 [9-31,34-38] were indexed in Scopus and were used as seed reports for citation searching; the remaining 2 [32,33] were not indexed in Scopus. Forward citation searching identified 321 records, and backward citation searching identified 621 records, yielding 942 records in total. After removal of 18 duplicates within the citation set and 146 records already identified through the database searches, the remaining 778 records were excluded during title and abstract screening. Citation searching identified no additional eligible studies (Figure 1). Ultimately, 30 studies [9-38] were included in the systematic review. Among these, 9 studies [25-31,37,38] were excluded from the quantitative synthesis because of unavailable extractable data, resulting in 21 studies [9-24,32-36] being included in the meta-analysis. Table S1 in Multimedia Appendix 2 [9-38] summarizes the characteristics of the included studies. The 30 studies [9-38] included in the systematic review were published between 2019 and 2026 [9-38]. Among the 21 studies [9-24,32-36] included in the meta-analysis, 18 were retrospective [10-24,32,33,36], 2 were prospective [9,35], and 1 [34] was a public dataset–based diagnostic model study. A total of 21 datasets [9-24,32-36] were extracted from the included studies. The number of datasets contributed by each study and the characteristics of the included datasets, including the institution or data source, data collection period, study population, assessment of population overlap, and basis for the independence judgment, are summarized in Table S2 in Multimedia Appendix 2. Stratified by validation strategy, there were 15 internal [9-13,15-20,22,24,34,35] and 6 external validation datasets [14,21,23,32,33,36]. In terms of imaging modality, 9 datasets used X-ray [11-13,15,17,18,32-34], 11 used MRI [9,10,14,16,19-23,35,36], and one used CT [24]. In terms of algorithms, the datasets evaluated 19 DL models [9-15,17-24,32-34,36] and 2 TML models [16,35]. Of the 19 DL datasets, 15 (78.9%) used convolutional neural networks (CNN) [11-13,15,17-22,24,32-34,36], one (5.3%) used a CNN-transformer hybrid model [14], one (5.3%) adopted a multilayer perceptron [9], and 2 (10.5%) used Transformer-based architectures [10,23] (Figure 2). Within the CNN subcategory, custom CNNs [13,17,22], Visual Geometry Group Network (VGGNet) [15,19,33], and Residual Network (ResNet) [11,24,32] each accounted for 15.8% (3/19), EfficientNet accounted for 10.5% (2/19), and densely connected convolutional network (DenseNet), Faster R-CNN, You Only Look Once (YOLO), and no-new U-Net (nnUNet) each accounted for 5.3% (1/19). Within the Transformer subcategory, Vision Transformer (ViT) and Swin Transformer each accounted for 5.3% (1/19). Within the CNN-transformer hybrid and MLP categories, ResNet-Transformer and deep neural network (DNN) each accounted for 5.3% (1/19), respectively.

**Figure 1.**
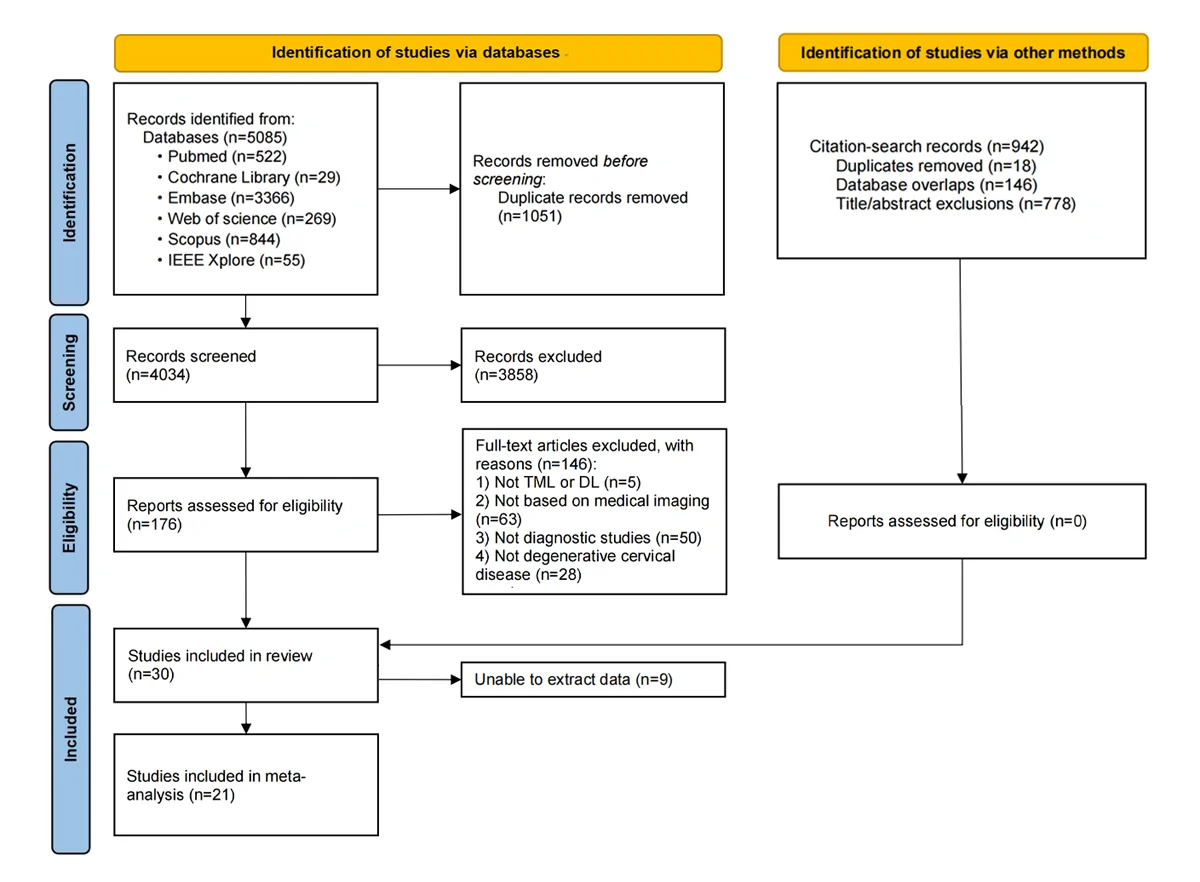
PRISMA (Preferred Reporting Items for Systematic Reviews and Meta-Analyses) flow diagram of the literature search strategy.

**Figure 2.**
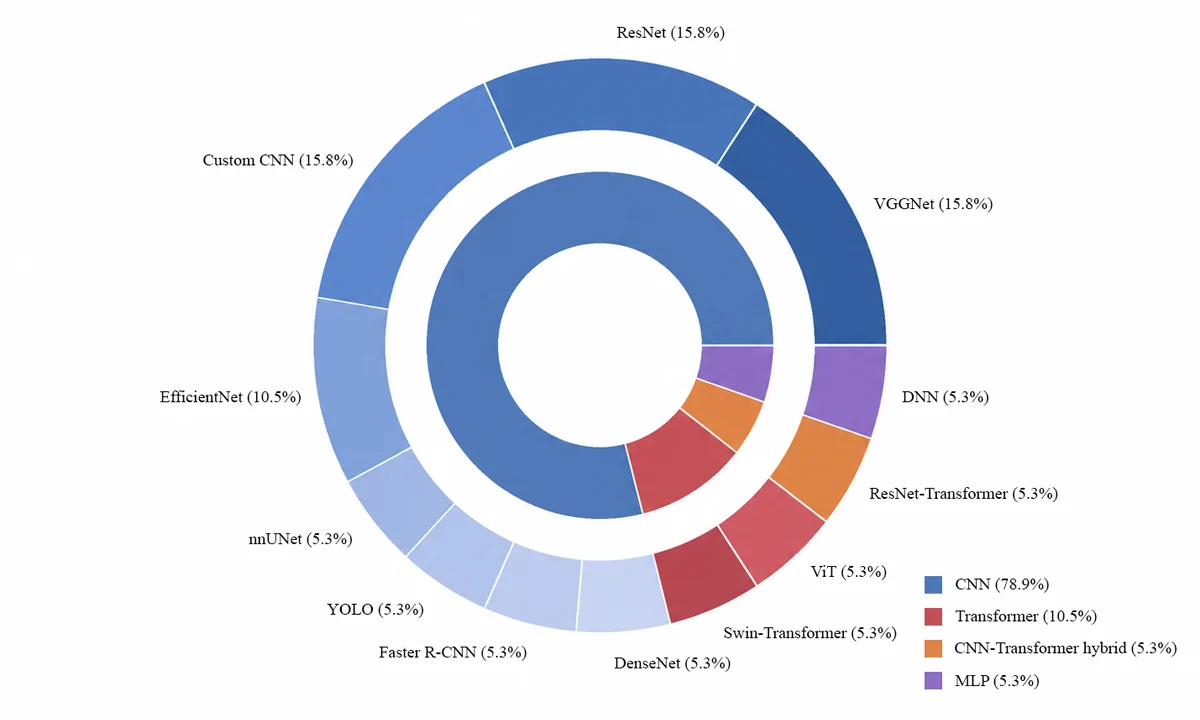
Distribution of model architectures used for the diagnosis of cervical degenerative diseases, including major categories and subcategories. CNN: convolutional neural network; DenseNet: densely connected convolutional network; DNN: deep neural network; nnUNet: no-new U-Net; R-CNN: region-based convolutional neural network; ResNet: Residual Network; VGGNet: Visual Geometry Group Network; ViT: Vision Transformer; YOLO: You Only Look Once.

### Risk of Bias in Studies

With regard to the methodological quality evaluated using the QUADAS-2 tool, Figure 3A and B presents the proportions of risk of bias across all QUADAS-2 domains and concerns regarding applicability across 3 domains and the summary of risk of bias and applicability concerns for each study according to QUADAS-2, respectively. Green, yellow, and red circles indicate low, unclear, and high risk of bias, respectively. Four studies [9,12,34,35] were identified as having a high risk of bias, 3 [20,22,32] having an unclear risk of bias, and 14 [10,11,13-19,21,23,24,33,36] having a completely low risk of bias. Four [9,12,34,35] of the included studies did not avoid a case-control study design, which resulted in a high risk of bias in patient selection. One study [22] did not clearly state whether consecutive or random sampling was used, resulting in an unclear risk of bias in patient selection. One study [9] showed high concerns regarding the applicability of patient selection owing to questionable population representativeness. One study [9] had overlapping training and test sets, resulting in a high risk of bias and high applicability concerns for the index test. One study derived cases and controls from different public datasets with different reference-standard or label sources, resulting in a high risk of bias in flow and timing [34]. Three additional studies did not report the interval between the index test and the reference standard, resulting in an unclear risk of bias in patient flow and timing [12,20,32].

With regard to the methodological quality evaluated using the QUADAS-AI tool, Figure 3C and D presents the proportions of risk of bias across all QUADAS-AI domains and the summary of risk of bias for each study according to QUADAS-AI, respectively. Most studies were at high risk of bias, primarily because of limited external validation, incomplete reporting of imaging acquisition details, and insufficient information for assessing dataset representativeness. Specifically, 17 studies [9-13,15-20,22,24,33-36] were identified as having a high risk of bias, one having an unclear risk of bias [32], and 3 [14,21,23] having a completely low risk of bias. Eight studies [9,11-13,15,19,33,36] did not provide the scanner model information used to acquire imaging data, resulting in a high risk of bias in patient selection. One study did not clearly state whether consecutive or random sampling was used, resulting in an unclear risk of bias in patient selection. Fifteen studies [9-13,15-20,22,24,34,35] did not use external validation, resulting in a high risk of bias for the index test. The risks of bias for the reference standard and flow and timing were consistent with those reported in the QUADAS-2 assessment above.

**Figure 3.**
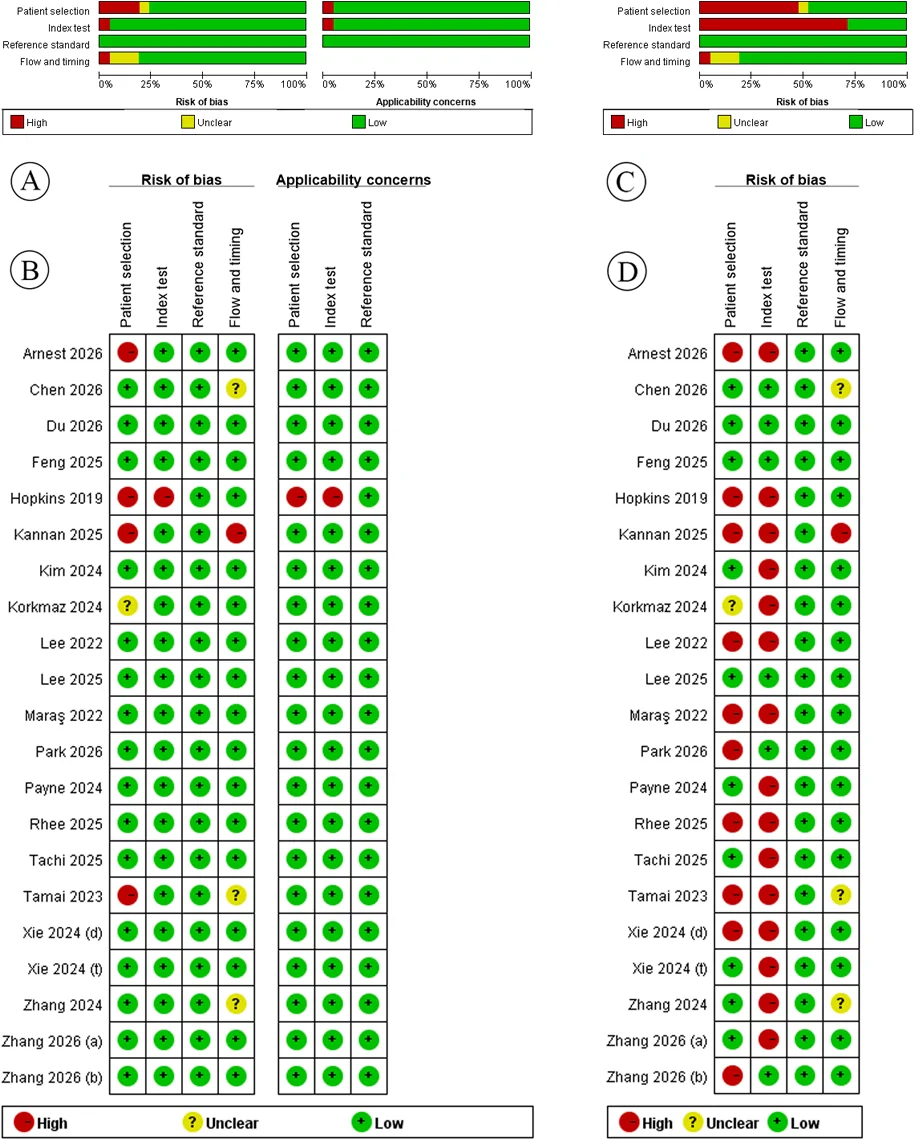
Methodological quality assessment based on the Quality Assessment of Diagnostic Accuracy Studies-2 (QUADAS-2) and the Quality Assessment of Diagnostic Accuracy Studies-Artificial Intelligence (QUADAS-AI) [9-24,32-36].

### Results of Syntheses

A total of 21 studies involving 25,301 patients reported the diagnostic performance of TML and DL models for cervical degenerative diseases (Figure 4). The Spearman correlation coefficient between the log sensitivity and log (1-specificity) was -0.168 (*P*=.46). The pooled sensitivity was 0.92 (95% CI 0.89‐0.96; *I*^2^=93.15%), with a 95% PI of 0.80‐1.00. The pooled specificity was 0.88 (95% CI 0.84‐0.91; *I*^2^=95.65%), with a 95% PI of 0.72‐1.00. The SROC curve showed an area under the curve of 0.96 (95% CI 0.94‐0.97) for TML and DL models in the diagnosis of cervical degenerative diseases, indicating a high diagnostic value (Figure 5).

**Figure 4.**
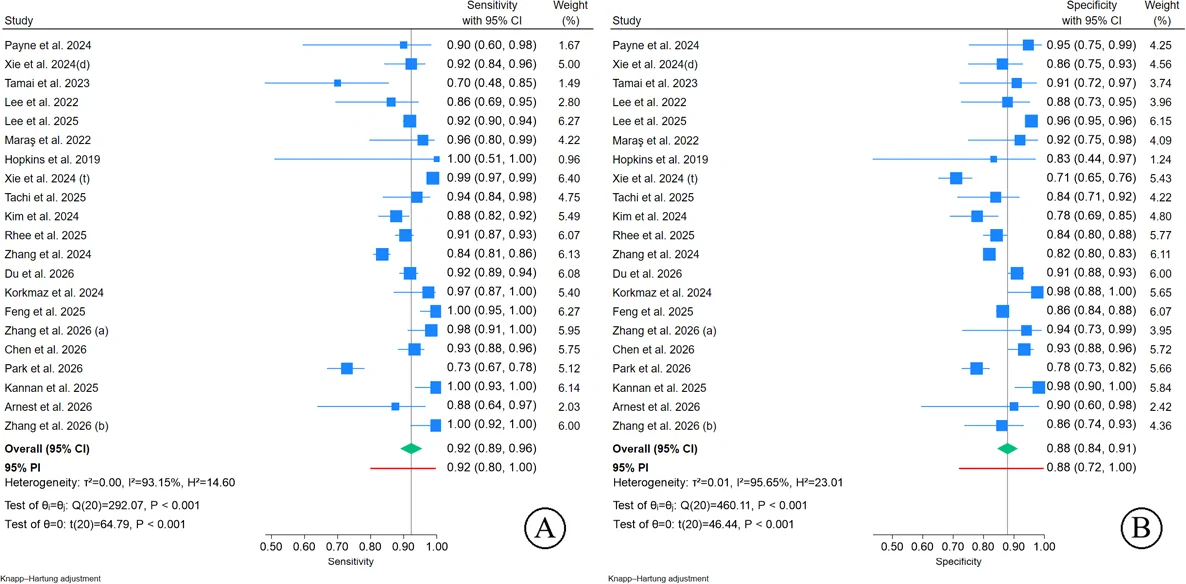
(A) Forest plot of sensitivity for TML and DL models in the diagnosis of cervical degenerative diseases. Individual study estimates are shown with 95% CI. In the random-effects meta-analysis using Knapp-Hartung adjustment, the pooled sensitivity was 0.92 (95% CI 0.89‐0.96). The vertical reference line represents the pooled sensitivity estimate. *I*^2^ was 93.15% (*P*<.001), and the 95% prediction interval was 0.80‐1.00. (B) Forest plot of specificity for TML and DL models in the diagnosis of cervical degenerative diseases. Individual study estimates are shown with 95% CI. In the random-effects meta-analysis using Knapp-Hartung adjustment, the pooled specificity was 0.88 (95% CI=0.84‐0.91). The vertical reference line represents the pooled specificity estimate. *I*^2^ was 95.65% (*P*<.001), and the 95% prediction interval was 0.72‐1.00 [9-24,32-36]. DL: deep learning; TML: traditional machine learning.

**Figure 5.**
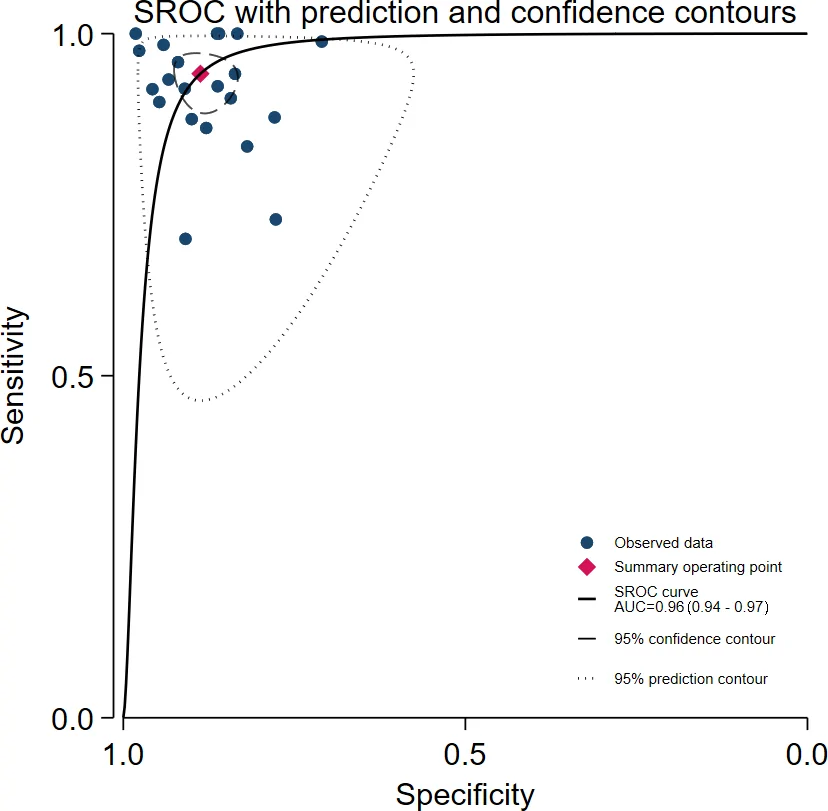
Summary receiver operating characteristic curve (SROC) for TML and DL models in the diagnosis of cervical degenerative diseases. Observed data points from individual studies, the summary operating point, and the summary receiver operating characteristic curve are shown. The AUC was 0.96 (95% CI 0.94‐0.97). The 95% confidence and prediction contours indicate the uncertainty around the summary estimate and the extent of between-study heterogeneity, respectively [9-24,32-36]. AUC: area under the curve; DL: deep learning; TML: traditional machine learning.

The posttest probability curves illustrated how test results transform different pretest probabilities (0%‐100%) into their corresponding posttest probabilities (Figure 6). Overall, the positive curve (LR^+^=8.36, 95% CI 6.14‐11.36) markedly increased the disease probability across the entire pretest range, whereas the negative curve (LR^-^=0.07, 95% CI 0.04‐0.11) markedly reduced it, both demonstrating good diagnostic performance. A pretest probability of 50% was used in the Fagan nomogram. At this pretest probability, a positive result from a TML or DL model increased the posttest probability of cervical degenerative disease to 89%, whereas a negative result reduced it to 6% (Figure 7). Across all cases, the unconditional positive predictive value was 0.81 (95% CI 0.78‐0.84), whereas the negative predictive value was 0.86 (95% CI 0.84‐0.89). The pooled LRs for TML and DL fell within the left lower quadrant (LR^+^<10, LR^-^<0.1) (Figure 8). The findings suggest that although the models achieved an acceptable overall performance and could help rule out cervical degenerative diseases, they remained insufficient to reliably diagnose them.

Leave-one-out sensitivity analyses showed that exclusion of any single study did not materially alter the pooled sensitivity or specificity. The pooled sensitivity ranged from 0.918 to 0.935, with corresponding 95% CI ranging from 0.884‐0.952 to 0.908‐0.964. The pooled specificity ranged from 0.872 to 0.888, with corresponding 95% CI ranging from 0.838‐0.906 to 0.858‐0.919. These findings indicate that no individual study had a substantial influence on the overall results and support the robustness of the main findings.

**Figure 6.**
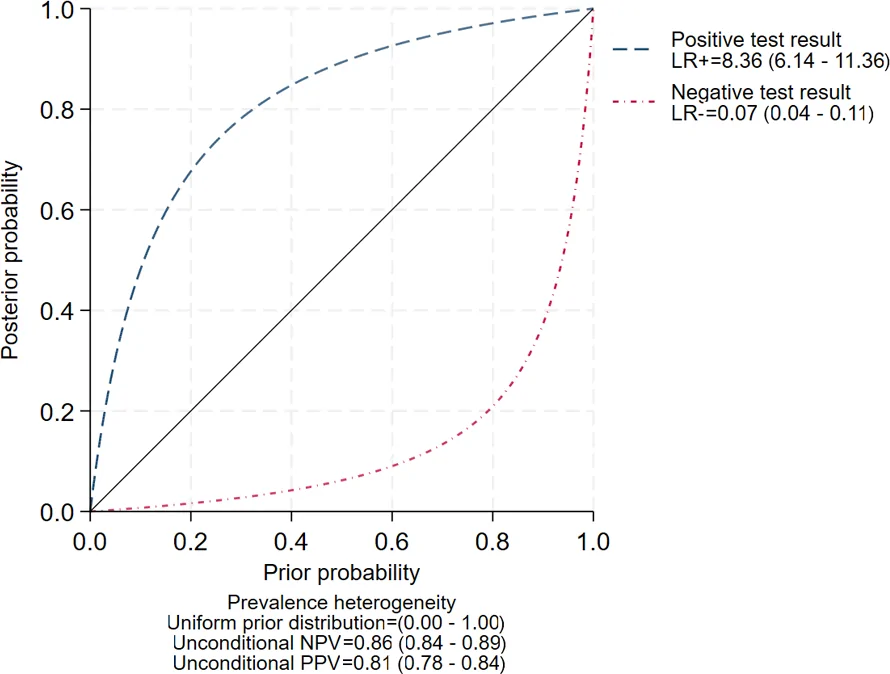
Posttest probability curves for TML and DL models in the diagnosis of cervical degenerative diseases. The blue dashed curve represents the posterior probability after a positive test result, and the red dotted curve represents the posterior probability after a negative test result, across a full range of prior probabilities. Based on the pooled likelihood ratios from the bivariate mixed-effects model, the positive likelihood ratio was 8.36 (95% CI 6.14‐11.36) and the negative likelihood ratio was 0.07 (95% CI 0.04‐0.11). These results indicate that a positive test result substantially increases the probability of disease, whereas a negative test result markedly decreases it. The unconditional NPV was 0.86 (95% CI 0.84‐0.89), and the unconditional PPV was 0.81 (95% CI 0.78‐0.84). DL: deep learning; LR: likelihood ratio; NPV: negative predictive value; PPV: positive predictive value; TML: traditional machine learning.

**Figure 7.**
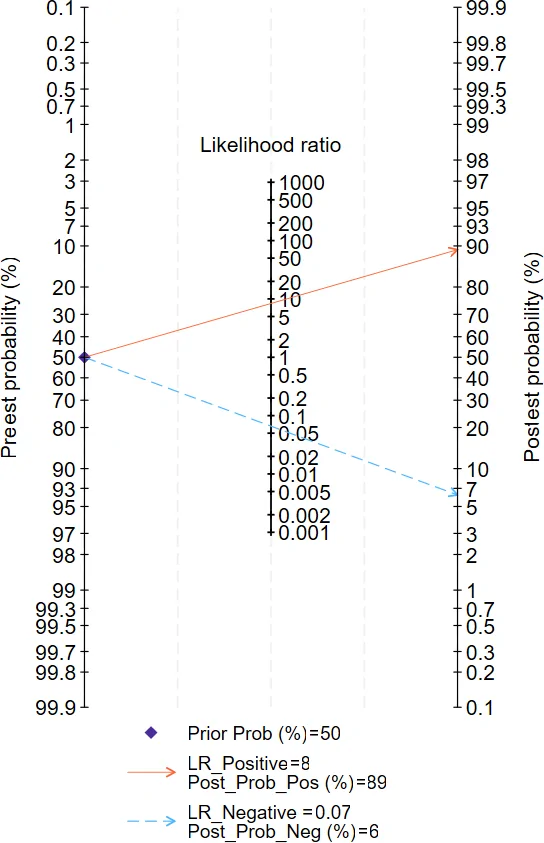
Fagan nomogram for TML and DL models in the diagnosis of cervical degenerative diseases. Assuming a pretest probability of 50%, a positive test result increased the posttest probability to 89%, whereas a negative test result decreased it to 6%. These changes were based on a pooled positive LR of 8 and a pooled negative LR of 0.07 derived from the bivariate mixed-effects model. DL: deep learning; LR: likelihood ratio; TML: traditional machine learning.

**Figure 8.**
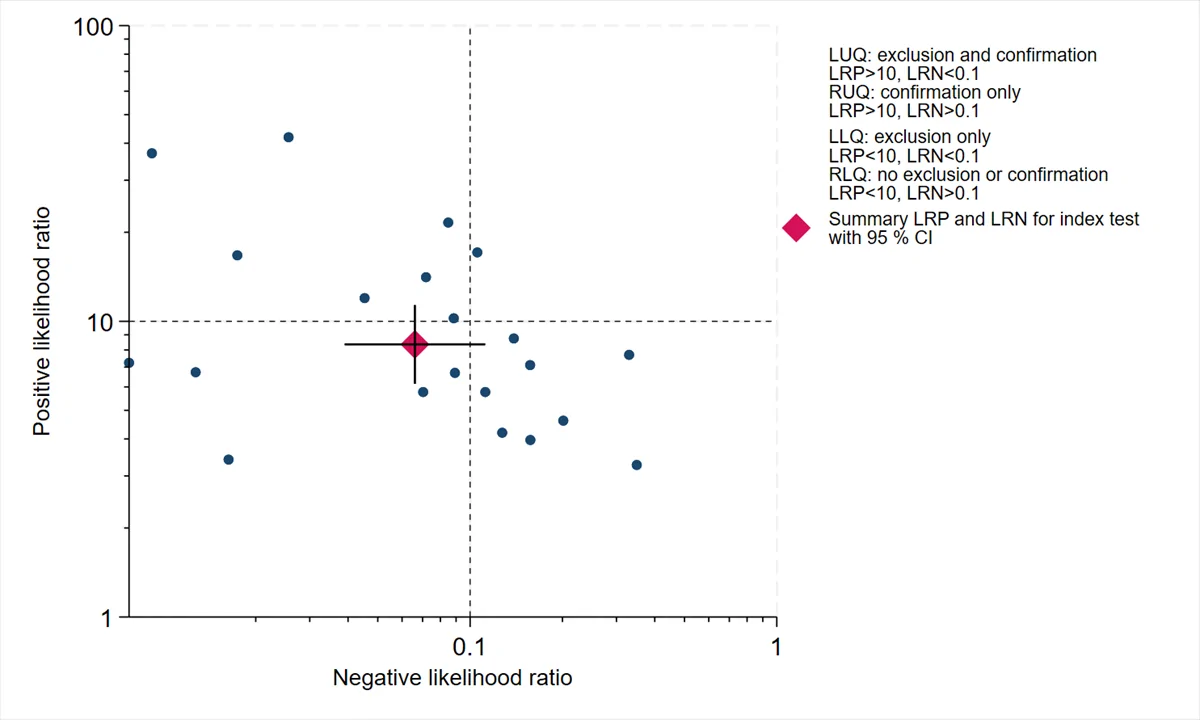
Likelihood-ratio scattergram for TML and DL models in the diagnosis of cervical degenerative diseases. Individual study estimates are shown as blue circles, and the pooled positive and negative likelihood ratios are shown as the red diamond with 95% CI bars. The pooled positive likelihood ratio was 8.36 (95% CI 6.14‐11.36), and the pooled negative likelihood ratio was 0.07 (95% CI 0.04‐0.11). The summary point was located in the left lower quadrant, where negative likelihood ratios are below 0.1 but positive likelihood ratios remain below 10, indicating strong rule-out performance but less definitive rule-in performance. DL: deep learning; LLQ: left lower quadrant; LRN: negative likelihood ratio; LRP: positive likelihood ratio; LUQ: left upper quadrant; RLQ: right lower quadrant; RUQ: right upper quadrant; TML: traditional machine learning.

Subgroup analyses were conducted across 2 domains, specifically validation strategy (internal vs external testing) and imaging modality (MRI vs X-ray). CT was not included in the imaging modality subgroup analysis because only one CT dataset was available, which did not meet the minimum number of datasets required for subgroup analysis. These analyses aimed to identify potential sources of heterogeneity and to elucidate how these factors influence the models’ diagnostic performance for cervical degenerative diseases (Table 1). Among the included datasets, the overall specificity of internal testing was lower than that of external testing (*P*<.001). MRI exhibited higher specificity than X-ray (*P*<.001).

For the bivariate boxplot (Figure 9), 4 floating points were out of the circles, suggesting variation in diagnostic performance across the included datasets.

**Table 1.** Results of subgroup analysis.

Categories	Studies, n	Sensitivity (95% CI)	*P* value (HBG^a^ of sensitivity)	Specificity (95% CI)	*P* value (HBG of specificity)
Validation			.06		<.001
Internal test	15 [9-13,15-20,22,24,34,35]	0.94 (0.90‐0.98)		0.88 (0.83‐0.92)	
External test	6 [14,21,23,32,33,36]	0.94 (0.89‐0.99)		0.90 (0.85‐0.95)	
Image			.17		<.001
X-ray	9 [11-13,15,17,18,32-34]	0.91 (0.85‐0.97)		0.88 (0.83‐0.94)	
MRI^b^	11 [9,10,14,16,19-23,35,36]	0.95 (0.92‐0.99)		0.89 (0.84‐0.93)	

a HBG: heterogeneity between groups.

b MRI: magnetic resonance imaging.

**Figure 9.**
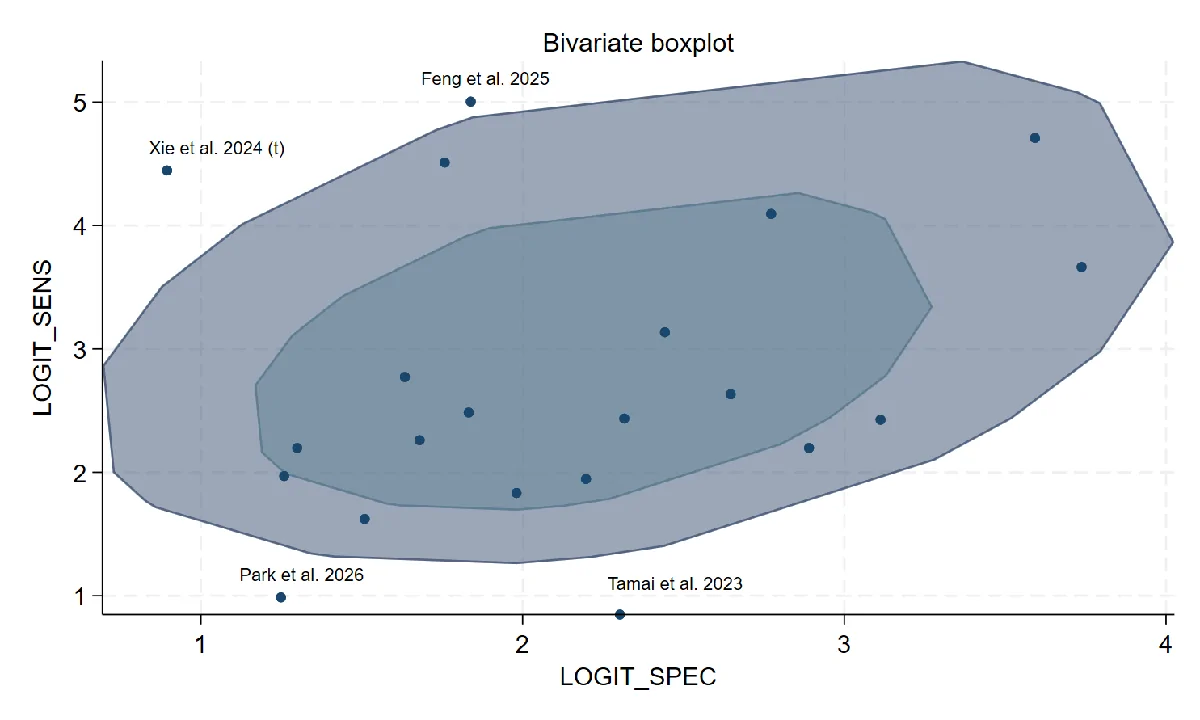
Bivariate boxplot of logit-transformed sensitivity and specificity for TML and DL models in the diagnosis of cervical degenerative diseases. Each point represents an individual study. The inner shaded region contains the central 50% of studies, and the outer shaded region represents the 95% confidence region. Studies located outside the outer region may indicate potential outliers or important between-study heterogeneity. Four studies were positioned outside the outer region, suggesting variation in diagnostic performance across the included datasets [12,16,23,33].

### Reporting Biases and Certainty of Evidence

Deeks funnel plot asymmetry test (Figure 10) revealed no significant evidence of publication bias or small-study effects (*P*=.35). According to the GRADE approach for diagnostic test accuracy studies, the overall certainty of evidence for the pooled diagnostic performance of TML and DL models was low. This rating reflected downgrading for serious risk of bias and serious inconsistency. The risk-of-bias judgment reflected the high QUADAS-AI risk assessments and incomplete reporting in some studies of data provenance and whether external datasets were fully separated from model training and hyperparameter tuning. The between-study heterogeneity and wide prediction intervals indicated inconsistency across study settings. No downgrading was applied for indirectness, imprecision, or publication bias.

**Figure 10.**
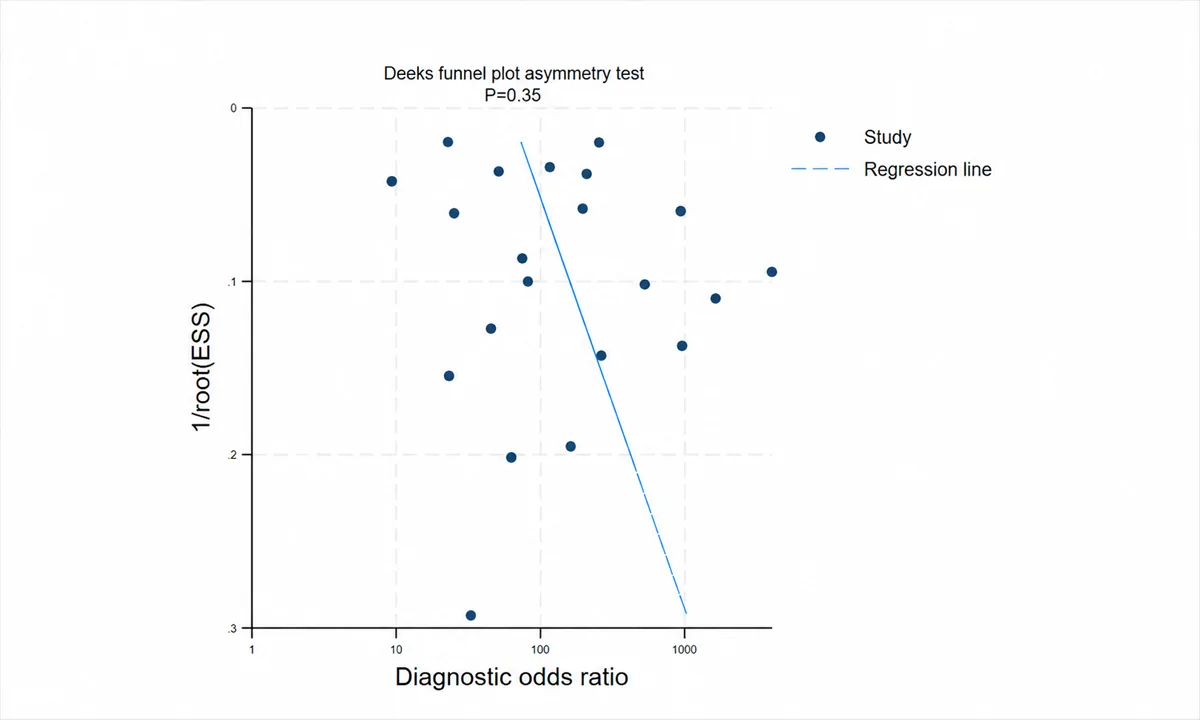
Deeks funnel plot asymmetry test for studies of TML and DL models in the diagnosis of cervical degenerative diseases. Each point represents an individual study, and the solid line represents the regression line. The *P* value of .35 indicates no significant evidence of publication bias or small-study effects. DL: deep learning; ESS: Effective Sample Size; TML: traditional machine learning.

## Discussion

### Principal Findings

This systematic review and meta-analysis comprehensively evaluated the diagnostic performance of TML and DL models for cervical degenerative diseases based on medical imaging. The pooled estimates provide disease-specific quantitative evidence supporting the ability of TML or DL models to diagnose cervical degenerative disease under experimental conditions.

The QUADAS-2, QUADAS-AI, and GRADE findings add important context to these diagnostic performance estimates. Although QUADAS-2 addresses conventional sources of bias in diagnostic accuracy studies, QUADAS-AI highlights AI-specific concerns, particularly limited external validation, incomplete reporting of data sources, imaging acquisition metadata and preprocessing procedures, and risks related to dataset splitting, sample size, class balance, and overfitting [51,52]. The risk of bias identified in the included studies and the observed between-study variation contributed to the low certainty of evidence in the GRADE assessment [53]. Therefore, the apparently favorable diagnostic performance should be interpreted alongside these methodological concerns rather than as evidence of immediate clinical readiness.

The relatively narrow CIs indicate that the pooled estimates were reasonably precise. However, sensitivity and specificity estimates varied across studies. The high *I*^2^ values suggest that much of this variation reflects differences in underlying diagnostic performance between studies rather than sampling error [54]. The PIs show the extent of this variation across comparable study settings, particularly for specificity [54]. The Spearman correlation coefficient and the distribution of SROC points did not indicate an evident threshold effect, and no characteristic shoulder pattern was observed between sensitivity and specificity, suggesting that differences in diagnostic thresholds were unlikely to be the primary source of the observed variation. Other potential sources were therefore explored using subgroup analyses and boxplot evaluation.

Subgroup analysis revealed that, regarding validation strategies, the overall specificity obtained from externally validated datasets was higher than that from internally validated ones. This finding is inconsistent with the commonly observed performance decline in external validation settings and should therefore be interpreted with caution [55]. To determine whether external test data were independent of training data and to exclude data leakage, we compared the institutions or data sources, collection periods, and study populations of the internal and external datasets (Table S3 in Multimedia Appendix 2). External test datasets were generally collected from institutions or data sources distinct from those of the corresponding internal development datasets, and no clear evidence of overlap or data leakage was identified. However, some studies did not fully report collection periods or explicitly state whether external datasets were excluded from all model training and hyperparameter tuning. First, the number of externally validated datasets included in this meta-analysis was relatively small (n=6) [14,21,23,32,33,36], compared with a substantially larger number of internally validated datasets. This imbalance may reduce the reliability of the subgroup estimate because random-effects inference may be less reliable when only a small number of studies are available [56,57]. Second, differences in dataset characteristics may contribute to this phenomenon. External validation datasets may include more clearly defined cases or higher-quality imaging, which can make classification easier and lead to higher estimated performance [55,58]. In the study by Lee et al [14], the external validation cohort appeared to contain a higher proportion of severe stenosis cases among abnormal images, where imaging features were more pronounced and easier for the model to identify. It leads to an overestimation of diagnostic performance, resulting in higher observed sensitivity and specificity compared with internal validation [14].

Among imaging modalities, MRI exhibited higher specificity but similar sensitivity to X-ray. MRI offers advantages in detecting soft-tissue abnormalities and early compression [59]. The diagnostic sensitivity of MRI is constrained by slice thickness and spatial resolution. Excessive slice thickness or inadequate spatial resolution hinders the reliable detection of small cervical spine lesions, thereby reducing diagnostic yield [60]. Contrarily, although plain radiography allows direct visualization of osseous structures, its inherently low soft-tissue contrast renders it susceptible to false-negative findings in the evaluation of cervical spondylosis [61].

In the comparison between TML and DL, this review only included 2 studies [16,35] that used TML as a classifier to distinguish images of cervical degenerative diseases from those of healthy controls. Therefore, no conclusion can be drawn regarding the diagnostic performance of TML versus DL for cervical degenerative diseases [16,35]. TML models required manual segmentation and demonstrated generally poorer performance than DL [62]. TML heavily depends on handcrafted features, which are time-consuming to design and may fail to capture subtle disease-related patterns [63]. Consistent with this limitation, Du et al [42] reported variable performance among TML models, mainly because of small sample sizes and the subjectivity involved in feature selection. DL can directly learn complex visual features from raw images, a capability that is essential for identifying fine pathological changes that are difficult to manually quantify [44]. However, the black-box nature of DL remains one of the main barriers to clinical adoption [64]. The majority of the current studies lack sufficient interpretability analyses, which limits clinicians’ trust in model decisions [65]. Future research must prioritize addressing the interpretability challenge by actively adopting advanced explainable AI techniques, such as gradient-weighted Class Activation Mapping (Grad-CAM) and Shapley Additive Explanations (SHAP) [66,67]. This approach can build clinician trust while helping clinicians and algorithm engineers quickly identify the potential causes of model errors [68]. Furthermore, as reported by Xie et al [16], the hybrid strategy of TML combined with DL exhibits strong potential. Precise segmentation performed by the DL component reduces the dimensionality of data entered into the TML classifier, thereby alleviating the “curse of dimensionality” commonly encountered by TML when handling high-dimensional data [65]. Concurrently, the interpretability of TML classification helps avoid the DL-associated black-box effect [16,69].

As regards differences among DL models, current evidence has not shown clear superiority of any specific architecture in the assessment of different cervical degenerative diseases or across different imaging analyses. Several studies have compared various DL models in terms of diagnostic performance for cervical spine disorders and have explored potential reasons for the observed differences [15,19,30]. For CNNs, ResNet and DenseNet tend to more heavily depend on large-scale training datasets [70]. Under small-sample training conditions, VGGNet may achieve better diagnostic performance owing to its relatively simple hierarchical stacking structure [15,71]. As regards interpretability, Rhee et al [19] used Grad-CAM to analyze the interpretability of different DL models in the diagnosis of cervical degenerative diseases. Their findings indicated that EfficientNet and MobileNet showed better interpretability, whereas ResNet and VGGNet demonstrated poorer performance. Although similar studies have reported minor differences in interpretability assessments across models, most results consistently indicated that EfficientNet offers superior interpretability [72,73]. EfficientNet uses a compound scaling strategy, enabling effective capture of high-level semantic features while maintaining anatomically reasonable heatmap distributions [74]. This characteristic may explain its enhanced interpretability [75].

The bivariate boxplot further illustrated between-study variation in diagnostic performance, with several datasets located outside the prediction region [12,16,23,33]. These outlier datasets suggest that variations in input strategies, model architectures, disease categories, and feature representation may contribute to the observed variability in diagnostic performance across studies.

A long-standing question is whether TML or DL models can outperform human clinicians in terms of diagnostic accuracy. In direct comparisons, several studies have reported that DL models achieve accuracy comparable to or even surpassing that of experienced spine specialists while providing considerably faster interpretation [11,12,14,20]. Previous studies have shown that DL models can effectively extract implicit features, which may be difficult for human observers to discern, thereby improving the robustness of segmentation [76]. However, in complex multiclass tasks, subtle calcification detection, and early-stage disease assessment, DL is still inferior to senior clinicians [29]. Therefore, TML or DL models are currently more suitable as assistive decision-support tools under strict supervision rather than independent replacements for human interpretation [77,78]. Their rapid interpretation capability may facilitate the rapid triage of suspected cervical cord compression cases [78]. For junior clinicians or residents with limited experience, TML or DL may provide second-reader support and reduce human errors or oversights through diagnostic accuracy comparable to that of senior experts [78]. Nevertheless, in complex clinical scenarios, including subtle early-stage lesions and postoperative spine imaging affected by metal implant artifacts, experienced radiologists still retain irreplaceable advantages [29]. Future studies should prioritize multicenter external validation and clinically representative datasets to facilitate the real-world implementation of AI-assisted diagnostic systems.

### Limitations

This review and meta-analysis has several limitations. First, among studies using DL models included in the meta-analysis, only 10 included training datasets with >1000 images [10,16,18-21,23,24,33,36]. DL algorithms typically require large volumes of high-quality data. Insufficient data may result in overfitting [79,80]. Cho et al [81] suggested that thousands of samples may be necessary to achieve extremely high diagnostic accuracy. Second, few studies used external validation, which limits the assessment of model generalizability. Third, it is important to note that the diagnosis of cervical degenerative diseases should not solely rely on imaging findings. In clinical practice, a definitive diagnosis requires a comprehensive evaluation in which radiological evidence must correlate with the patient’s clinical symptoms and physical signs [4]. As this study exclusively focused on the performance of AI models in analyzing imaging data, it did not account for the integration of clinical information, which is crucial for a holistic diagnosis. Fourth, only peer-reviewed English language studies were included in the final synthesis. This restriction may have introduced language bias and reduced the completeness and timeliness of the evidence base. As a result, findings from non–English speaking settings and recent unpublished work may be underrepresented in this review.

### Conclusions

This systematic review and meta-analysis provides a quantitative synthesis of the diagnostic performance of TML and DL for cervical degenerative diseases across MRI, X-ray, and CT. Compared with previously published reviews in this field, it pools diagnostic performance estimates and evaluates uncertainty across studies. The pooled results showed satisfactory diagnostic performance, suggesting that these models may assist in the diagnosis of cervical degenerative diseases. However, most included studies were retrospective and conducted at a single center, and external validation was uncommon. Between-study heterogeneity and low certainty of evidence further limit confidence in model performance in routine clinical settings. At present, AI should be used to support, rather than replace, physician interpretation and clinical decision-making.

## Supplementary material

10.2196/92008 Multimedia Appendix 1 Full search strings.

10.2196/92008 Multimedia Appendix 2 Characteristics of studies included in the systematic review and assessments of dataset independence.

10.2196/92008 Checklist 1 PRISMA 2020 for Abstracts checklist.

10.2196/92008 Checklist 2 PRISMA 2020 checklist.

10.2196/92008 Checklist 3 PRISMA-S checklist.

## Abbreviations

CNN: convolutional neural network
CT: computed tomography
DenseNet: densely connected convolutional network
DL: deep learning
DNN: deep neural network
Grad-CAM: gradient-weighted class activation mapping
GRADE: Grading of Recommendations Assessment, Development and Evaluation
IEEE: Institute of Electrical and Electronics Engineers
LR: likelihood ratio
MIDAS: Meta-Analytical Integration of Diagnostic Accuracy Studies
ML: machine learning
MRI: magnetic resonance imaging
nnUNet: no-new U-Net
PI: prediction interval
PIT: participants, index tests, and target condition
PRISMA: Preferred Reporting Items for Systematic Reviews and Meta-Analyses
PRISMA-DTA: Preferred Reporting Items for a Systematic Review and Meta-analysis of Diagnostic Test Accuracy Studies
PRISMA-S: Preferred Reporting Items for Systematic Reviews and Meta-Analyses literature search extension
QUADAS-2: Quality Assessment of Diagnostic Accuracy Studies 2
QUADAS-AI: Quality Assessment of Diagnostic Accuracy Studies AI
ResNet: Residual Network
SHAP: Shapley Additive Explanations
SROC: summary receiver operating characteristic
TML: traditional machine learning
VGGNet: Visual Geometry Group Network
ViT: Vision Transformer
YOLO: You Only Look Once
