# Supplementary material for: Machine Learning and Deep Learning for the Diagnosis of Cervical Degenerative Diseases: Systematic Review and Meta-Analysis
Source: J Med Internet Res. 2026 Oct 5;28:e92008. doi: 10.2196/92008 (PMC13637981; doi:10.2196/92008)
Supplement: Supplementary file 1 [file jmir-v28-e92008-s001.docx]

**Table S1. PIT/PIRD framework, PICO crosswalk, and implementation in the search strategy**

| **Framework element** | **Definition in this review** | **Search implementation** | **Mandatory search concept** |
| --- | --- | --- | --- |
| P / Population | Adults undergoing cervical spine imaging with suspected or confirmed degenerative cervical disease. | Population was operationalized through the cervical target-condition block; no separate demographic term was required because age and clinical status are often absent from titles and abstracts. | No separate block |
| I / Index test | Traditional machine-learning or deep-learning models used for imaging-based diagnosis, detection, classification, segmentation, or grading. | Controlled vocabulary and free-text terms for artificial intelligence, machine learning, deep learning, computer-assisted diagnosis/detection, automated image analysis, pattern recognition, radiomics, and related generic concepts. | Yes — AI/index-test concept group |
| T/D / Target condition or diagnosis of interest | Radiographically detectable cervical degenerative conditions, including cervical spondylosis, degenerative cervical myelopathy, cervical spinal/canal/foraminal stenosis, cervical cord compression, cervical disc degeneration/herniation, and cervical radiculopathy. | Controlled vocabulary (MeSH/Emtree where available) plus prospectively defined free-text synonyms for cervical degenerative disease entities. | Yes — disease concept group |
| R / Reference standard | Expert image interpretation, established imaging criteria, operative/clinical diagnosis, or another reference standard used by the primary study. | Not required as a search concept because reference standards are inconsistently described in searchable fields and would reduce sensitivity. | No |
| C / Comparator | Not required for this diagnostic-accuracy review; eligible studies could compare model output with a reference standard without naming a separate comparator group. | No comparator terms were required. | No |
| O / Diagnostic outcomes | Diagnostic-accuracy measures derived from or permitting reconstruction of 2 x 2 data, including sensitivity, specificity, likelihood ratios, and SROC/AUC. | Not required as a search concept to avoid missing studies that report diagnostic outcomes only in the full text. | No |

Rationale: This review addressed diagnostic test accuracy. PIT was used to define the review question. PIRD was used to present the reference standard as a separate component. PICO was included only as a reporting crosswalk and was not used to construct the search. The database searches required two concept blocks, the index test and the target condition. Terms for the population, reference standard, comparator, and diagnostic outcomes were not mandatory because inconsistent indexing or reporting of these concepts could reduce search sensitivity.

Search-design safeguards: The strategy combined controlled vocabulary with free-text terms, used exploded subject headings where supported, avoided outcome and study-design filters, and was constructed prospectively from the review question. Named architectures found in the included studies were not added retrospectively. The strategy was developed by HD and reviewed by LZ. It did not undergo formal external review using the Peer Review of Electronic Search Strategies (PRESS) guideline.

**Table S2. Database- and platform-specific search strategies exactly as executed**

| **Database** | **Search strategy** | **Filters and Limits** | **Number of Records** |
| --- | --- | --- | --- |
| PubMed  (pubmed.ncbi.nlm.nih.gov) | (  ("Spondylosis"[Mesh] AND "Cervical Vertebrae"[Mesh]) OR  ("Spinal Stenosis"[Mesh] AND "Cervical Vertebrae"[Mesh]) OR  ("Spinal Cord Compression"[Mesh] AND "Cervical Vertebrae"[Mesh]) OR  ("Intervertebral Disc Degeneration"[Mesh] AND "Cervical Vertebrae"[Mesh]) OR  ("Intervertebral Disc Displacement"[Mesh] AND "Cervical Vertebrae"[Mesh]) OR  ("Radiculopathy"[Mesh] AND "Cervical Vertebrae"[Mesh]) OR  ("cervical"[Title/Abstract] AND ("disc degeneration"[Title/Abstract] OR "disk degeneration"[Title/Abstract])) OR  "cervical spondylos*"[Title/Abstract] OR  "degenerative cervical myelopath*"[Title/Abstract] OR  "cervical spondylotic myelopath*"[Title/Abstract] OR  "cervical myelopath*"[Title/Abstract] OR  "degenerative cervical spondylos*"[Title/Abstract] OR  "cervical spinal stenos*"[Title/Abstract] OR  "cervical canal stenos*"[Title/Abstract] OR  "cervical central canal stenos*"[Title/Abstract] OR  "cervical foraminal stenos*"[Title/Abstract] OR  "cervical neural foraminal stenos*"[Title/Abstract] OR  "cervical spinal cord compression"[Title/Abstract] OR  "cervical cord compression"[Title/Abstract] OR  "cervical disc degenerat*"[Title/Abstract] OR  "cervical disk degenerat*"[Title/Abstract] OR  "cervical degenerative disc disease"[Title/Abstract] OR  "cervical degenerative disk disease"[Title/Abstract] OR  "cervical disc disease"[Title/Abstract] OR  "cervical disk disease"[Title/Abstract] OR  "cervical disc herniat*"[Title/Abstract] OR  "cervical disk herniat*"[Title/Abstract] OR  "cervical disc prolaps*"[Title/Abstract] OR  "cervical disk prolaps*"[Title/Abstract] OR  "cervical radiculopath*"[Title/Abstract] OR  "cervical spondylotic radiculopath*"[Title/Abstract] OR  "degenerative cervical spine"[Title/Abstract] OR  "cervical spine degenerat*"[Title/Abstract] OR  "cervical degenerative disease*"[Title/Abstract]  )  AND  (  "Artificial Intelligence"[Mesh] OR  "Machine Learning"[Mesh] OR  "Deep Learning"[Mesh] OR  "Neural Networks, Computer"[Mesh] OR  "Diagnosis, Computer-Assisted"[Mesh] OR  "Image Processing, Computer-Assisted"[Mesh] OR  "Pattern Recognition, Automated"[Mesh] OR  "Radiomics"[Mesh] OR  "artificial intelligence"[Title/Abstract] OR  "machine learning"[Title/Abstract] OR  "deep learning"[Title/Abstract] OR  "neural network*"[Title/Abstract] OR  "convolutional neural network*"[Title/Abstract] OR  "computer vision"[Title/Abstract] OR  "computer aided diagnos*"[Title/Abstract] OR  "computer assisted diagnos*"[Title/Abstract] OR  "computer aided detect*"[Title/Abstract] OR  "computer assisted detect*"[Title/Abstract] OR  "automated diagnos*"[Title/Abstract] OR  "automated detect*"[Title/Abstract] OR  "automated classif*"[Title/Abstract] OR  "automated segment*"[Title/Abstract] OR  "automated grad*"[Title/Abstract] OR  "image analys*"[Title/Abstract] OR  "image classif*"[Title/Abstract] OR  "image segment*"[Title/Abstract] OR  "pattern recogn*"[Title/Abstract] OR  "radiomic*"[Title/Abstract] OR  "texture analys*"[Title/Abstract]  )  AND  ("2000/01/01"[Date - Publication] : "2026/06/23"[Date - Publication]) | Searched June 23, 2026. Publication date: January 1, 2000, to June 23, 2026. No language, publication-type, or study-design filter. | 522 |
| Embase  (embase.com) | (  ('spondylosis'/exp AND 'cervical spine'/exp) OR  ('spinal stenosis'/exp AND 'cervical spine'/exp) OR  ('spinal cord compression'/exp AND 'cervical spine'/exp) OR  ('intervertebral disc degeneration'/exp AND 'cervical spine'/exp) OR  ('intervertebral disk hernia'/exp AND 'cervical spine'/exp) OR  ('radiculopathy'/exp AND 'cervical spine'/exp) OR  'cervical spondylos*':ti,ab,kw OR  'degenerative cervical myelopath*':ti,ab,kw OR  'cervical spondylotic myelopath*':ti,ab,kw OR  'cervical myelopath*':ti,ab,kw OR  'degenerative cervical spondylos*':ti,ab,kw OR  'cervical spinal stenos*':ti,ab,kw OR  'cervical canal stenos*':ti,ab,kw OR  'cervical central canal stenos*':ti,ab,kw OR  'cervical foraminal stenos*':ti,ab,kw OR  'cervical neural foraminal stenos*':ti,ab,kw OR  'cervical spinal cord compression':ti,ab,kw OR  'cervical cord compression':ti,ab,kw OR  'cervical disc degenerat*':ti,ab,kw OR  'cervical disk degenerat*':ti,ab,kw OR  'cervical degenerative disc disease':ti,ab,kw OR  'cervical degenerative disk disease':ti,ab,kw OR  'cervical disc disease':ti,ab,kw OR  'cervical disk disease':ti,ab,kw OR  'cervical disc herniat*':ti,ab,kw OR  'cervical disk herniat*':ti,ab,kw OR  'cervical disc prolaps*':ti,ab,kw OR  'cervical disk prolaps*':ti,ab,kw OR  'cervical radiculopath*':ti,ab,kw OR  'cervical spondylotic radiculopath*':ti,ab,kw OR  'degenerative cervical spine':ti,ab,kw OR  'cervical spine degenerat*':ti,ab,kw OR  'cervical degenerative disease*':ti,ab,kw OR  (cervical:ti,ab,kw AND ('disc degeneration':ti,ab,kw OR 'disk degeneration':ti,ab,kw))  )  AND  (  'artificial intelligence'/exp OR  'machine learning'/exp OR  'deep learning'/exp OR  'neural network'/exp OR  'computer assisted diagnosis'/exp OR  'computer assisted image interpretation'/exp OR  'image processing'/exp OR  'pattern recognition'/exp OR  'radiomics'/exp OR  'artificial intelligence':ti,ab,kw OR  'machine learning':ti,ab,kw OR  'deep learning':ti,ab,kw OR  'neural network*':ti,ab,kw OR  'convolutional neural network*':ti,ab,kw OR  'computer vision':ti,ab,kw OR  'computer aided diagnos*':ti,ab,kw OR  'computer assisted diagnos*':ti,ab,kw OR  'computer aided detect*':ti,ab,kw OR  'computer assisted detect*':ti,ab,kw OR  'automated diagnos*':ti,ab,kw OR  'automated detect*':ti,ab,kw OR  'automated classif*':ti,ab,kw OR  'automated segment*':ti,ab,kw OR  'automated grad*':ti,ab,kw OR  'image analys*':ti,ab,kw OR  'image classif*':ti,ab,kw OR  'image segment*':ti,ab,kw OR  'pattern recogn*':ti,ab,kw OR  'radiomic*':ti,ab,kw OR  'texture analys*':ti,ab,kw  )  AND [01-01-2000 to 23-06-2026]/pd | Searched June 23, 2026. Publication date: January 1, 2000, to June 23, 2026. No language, publication-type, or study-design filter. | 3,366 |
| Cochrane CENTRAL  (Cochrane Library Search Manager) | Controlled-vocabulary lines created through the MeSH panel (Look up -> Explode all trees -> Add/Edit search line; no subheadings/qualifiers):  #1 Spondylosis — Explode all trees  #2 Cervical Vertebrae — Explode all trees  #3 Spinal Stenosis — Explode all trees  #4 Spinal Cord Compression — Explode all trees  #5 Intervertebral Disc Degeneration — Explode all trees  #6 Intervertebral Disc Displacement — Explode all trees  #7 Radiculopathy — Explode all trees  #8 (#1 AND #2) OR (#3 AND #2) OR (#4 AND #2) OR (#5 AND #2) OR (#6 AND #2) OR (#7 AND #2)  #9 ((cervical NEXT spondylos*) OR (degenerative NEXT cervical NEXT myelopath*) OR (cervical NEXT spondylotic NEXT myelopath*) OR (cervical NEXT myelopath*) OR (degenerative NEXT cervical NEXT spondylos*) OR (cervical NEXT spinal NEXT stenos*) OR (cervical NEXT canal NEXT stenos*) OR (cervical NEXT central NEXT canal NEXT stenos*) OR (cervical NEXT foraminal NEXT stenos*) OR (cervical NEXT neural NEXT foraminal NEXT stenos*) OR (cervical NEXT spinal NEXT cord NEXT compression) OR (cervical NEXT cord NEXT compression) OR (cervical NEXT disc NEXT degenerat*) OR (cervical NEXT disk NEXT degenerat*) OR (cervical NEXT degenerative NEXT disc NEXT disease) OR (cervical NEXT degenerative NEXT disk NEXT disease) OR (cervical NEXT disc NEXT disease) OR (cervical NEXT disk NEXT disease) OR (cervical NEXT disc NEXT herniat*) OR (cervical NEXT disk NEXT herniat*) OR (cervical NEXT disc NEXT prolaps*) OR (cervical NEXT disk NEXT prolaps*) OR (cervical NEXT radiculopath*) OR (cervical NEXT spondylotic NEXT radiculopath*) OR (degenerative NEXT cervical NEXT spine) OR (cervical NEXT spine NEXT degenerat*) OR (cervical NEXT degenerative NEXT disease*) OR (cervical AND ((disc NEXT degeneration) OR (disk NEXT degeneration)))):ti,ab,kw  #10 #8 OR #9  #11 Artificial Intelligence — Explode all trees  #12 Machine Learning — Explode all trees  #13 Deep Learning — Explode all trees  #14 Neural Networks, Computer — Explode all trees  #15 Diagnosis, Computer-Assisted — Explode all trees  #16 Image Processing, Computer-Assisted — Explode all trees  #17 Pattern Recognition, Automated — Explode all trees  #18 Radiomics — Explode all trees  #19 ((artificial NEXT intelligence) OR (machine NEXT learning) OR (deep NEXT learning) OR (neural NEXT network*) OR (convolutional NEXT neural NEXT network*) OR (computer NEXT vision) OR (computer NEXT aided NEXT diagnos*) OR (computer NEXT assisted NEXT diagnos*) OR (computer NEXT aided NEXT detect*) OR (computer NEXT assisted NEXT detect*) OR (automated NEXT diagnos*) OR (automated NEXT detect*) OR (automated NEXT classif*) OR (automated NEXT segment*) OR (automated NEXT grad*) OR (image NEXT analys*) OR (image NEXT classif*) OR (image NEXT segment*) OR (pattern NEXT recogn*) OR radiomic* OR (texture NEXT analys*)):ti,ab,kw  #20 #11 OR #12 OR #13 OR #14 OR #15 OR #16 OR #17 OR #18 OR #19  #21 #10 AND #20 | Searched June 23, 2026. CENTRAL records; date coverage January 1, 2000, to June 23, 2026. No language filter. MeSH terms exploded; no qualifiers selected. | 29 |
| Web of Science Core Collection  (Query Builder) | TS=("cervical spondylos*" OR "degenerative cervical myelopath*" OR "cervical spondylotic myelopath*" OR "cervical myelopath*" OR "degenerative cervical spondylos*" OR "cervical spinal stenos*" OR "cervical canal stenos*" OR "cervical central canal stenos*" OR "cervical foraminal stenos*" OR "cervical neural foraminal stenos*" OR "cervical spinal cord compression" OR "cervical cord compression" OR "cervical disc degenerat*" OR "cervical disk degenerat*" OR "cervical degenerative disc disease" OR "cervical degenerative disk disease" OR "cervical disc disease" OR "cervical disk disease" OR "cervical disc herniat*" OR "cervical disk herniat*" OR "cervical disc prolaps*" OR "cervical disk prolaps*" OR "cervical radiculopath*" OR "cervical spondylotic radiculopath*" OR "degenerative cervical spine" OR "cervical spine degenerat*" OR "cervical degenerative disease*" OR (cervical AND ("disc degeneration" OR "disk degeneration")))  AND TS=("artificial intelligence" OR "machine learning" OR "deep learning" OR "neural network*" OR "convolutional neural network*" OR "computer vision" OR "computer aided diagnos*" OR "computer assisted diagnos*" OR "computer aided detect*" OR "computer assisted detect*" OR "automated diagnos*" OR "automated detect*" OR "automated classif*" OR "automated segment*" OR "automated grad*" OR "image analys*" OR "image classif*" OR "image segment*" OR "pattern recogn*" OR "radiomic*" OR "texture analys*")  AND DOP=2000-01-01/2026-06-23 | Searched June 23, 2026. DOP=2000-01-01/2026-06-23. All available Core Collection editions; no language or document-type restriction. | 269 |
| IEEE Xplore  (Advanced Search) | Row 1 — Field: All Metadata  "cervical spondylosis" OR "degenerative cervical myelopathy" OR "cervical spondylotic myelopathy" OR "cervical myelopathy" OR "cervical spinal stenosis" OR "cervical canal stenosis" OR "cervical central canal stenosis" OR "cervical foraminal stenosis" OR "cervical neural foraminal stenosis" OR "cervical spinal cord compression" OR "cervical cord compression" OR "cervical disc degeneration" OR "cervical disk degeneration" OR "cervical degenerative disc disease" OR "cervical degenerative disk disease" OR "cervical disc herniation" OR "cervical disk herniation" OR "cervical disc prolapse" OR "cervical disk prolapse" OR "cervical radiculopathy" OR "cervical spondylotic radiculopathy" OR "degenerative cervical spine" OR "cervical degenerative disease"  Row 2 — Operator: AND; Field: All Metadata  "artificial intelligence" OR "machine learning" OR "deep learning" OR "neural network" OR "neural networks" OR "convolutional neural network" OR "convolutional neural networks" OR "computer vision" OR "computer aided diagnosis" OR "computer assisted diagnosis" OR "computer aided detection" OR "computer assisted detection" OR "automated diagnosis" OR "automated detection" OR "automated classification" OR "automated segmentation" OR "automated grading" OR "image analysis" OR "image classification" OR "image segmentation" OR "pattern recognition" OR "radiomics" OR "texture analysis"  Publication Date: Publication Year, From 2000 to 2026 (not Date Added to IEEE Xplore). | Searched June 23, 2026. All Metadata fields; Publication Year 2000-2026; predefined June 23, 2026 cutoff checked during record assessment. No content-type, publisher, or open-access restriction. | 55 |
| Scopus  (Advanced document search) | TITLE-ABS-KEY("cervical spondylos*" OR "degenerative cervical myelopath*" OR "cervical spondylotic myelopath*" OR "cervical myelopath*" OR "degenerative cervical spondylos*" OR "cervical spinal stenos*" OR "cervical canal stenos*" OR "cervical central canal stenos*" OR "cervical foraminal stenos*" OR "cervical neural foraminal stenos*" OR "cervical spinal cord compression" OR "cervical cord compression" OR "cervical disc degenerat*" OR "cervical disk degenerat*" OR "cervical degenerative disc disease" OR "cervical degenerative disk disease" OR "cervical disc disease" OR "cervical disk disease" OR "cervical disc herniat*" OR "cervical disk herniat*" OR "cervical disc prolaps*" OR "cervical disk prolaps*" OR "cervical radiculopath*" OR "cervical spondylotic radiculopath*" OR "degenerative cervical spine" OR "cervical spine degenerat*" OR "cervical degenerative disease*" OR (cervical AND ("disc degeneration" OR "disk degeneration")))  AND TITLE-ABS-KEY("artificial intelligence" OR "machine learning" OR "deep learning" OR "neural network*" OR "convolutional neural network*" OR "computer vision" OR "computer aided diagnos*" OR "computer assisted diagnos*" OR "computer aided detect*" OR "computer assisted detect*" OR "automated diagnos*" OR "automated detect*" OR "automated classif*" OR "automated segment*" OR "automated grad*" OR "image analys*" OR "image classif*" OR "image segment*" OR "pattern recogn*" OR "radiomic*" OR "texture analys*")  AND PUBYEAR > 1999 AND PUBYEAR < 2027 | Searched June 23, 2026. TITLE-ABS-KEY; PUBYEAR 2000-2026; predefined June 23, 2026 cutoff checked during record assessment. No language, document-type, or subject-area restriction. | 844 |

Supplementary citation-searching method

On June 23, 2026, the 30 included studies were searched by DOI in Scopus; 28 indexed reports were available as seeds. Their cited references and citing documents were retrieved, combined, deduplicated by DOI and normalized title/year, compared with the six-database set, and screened using the same eligibility criteria. No registries, websites, organizations, or other supplementary sources were searched. Citation-record disposition is reported in Figure 1.

**Table S3. Exact Scopus DOI query used to locate the 30 seed reports for citation searching**

| **Purpose** | **Exact query** | **Outcome** |
| --- | --- | --- |
| Seed-report identification before forward and backward citation searching | DOI("10.1002/acm2.13001") OR DOI("10.1055/s-0044-1779317") OR DOI("10.1186/s12880-024-01489-w") OR DOI("10.52312/jdrs.2022.445") OR DOI("10.1007/s00586-025-08908-8") OR DOI("10.1016/j.spinee.2024.09.015") OR DOI("10.3390/s24113428") OR DOI("10.3390/bioengineering12060604") OR DOI("10.3174/ajnr.a8141") OR DOI("10.1038/s41467-026-69023-w") OR DOI("10.1186/s12883-022-02670-w") OR DOI("10.3349/ymj.2023.0091") OR DOI("10.1097/brs.0000000000004595") OR DOI("10.1038/s41598-021-89848-3") OR DOI("10.1097/brs.0000000000005639") OR DOI("10.1016/j.wneu.2019.03.165") OR DOI("10.1097/brs.0000000000005414") OR DOI("10.3389/fnins.2024.1501972") OR DOI("10.3389/fphys.2023.1281506") OR DOI("10.21037/qims-2025-67") OR DOI("10.1007/s00256-025-04917-2") OR DOI("10.3389/fneur.2021.711880") OR DOI("10.1007/s00586-023-07641-4") OR DOI("10.3171/2026.1.spine251409") OR DOI("10.1038/s41598-026-56657-5") OR DOI("10.29294/ijase.12.1.2025.4871-4884") OR DOI("10.1007/s00586-026-10043-x") OR DOI("10.1109/jbhi.2025.3598469") OR DOI("10.3390/electronics12010195") OR DOI("10.1109/access.2024.3432803") | 28 of 30 seed reports were indexed in Scopus; citation-flow counts are reported in Figure 1. |

Record-management note: Database records were managed in Zotero, and exact duplicates were identified by DOI and normalized title/year. The six databases yielded 5,085 records; 1,051 duplicates were removed, leaving 4,034 for screening. Citation searching was reported separately to avoid double counting database-retrieved records.
