# Supplementary material for: Machine Learning and Deep Learning for the Diagnosis of Cervical Degenerative Diseases: Systematic Review and Meta-Analysis
Source: J Med Internet Res. 2026 Oct 5;28:e92008. doi: 10.2196/92008 (PMC13637981; doi:10.2196/92008)
Supplement: Supplementary file 2 [file jmir-v28-e92008-s002.docx]

Table S1. Characteristics of studies included in the systematic review.

| Study | Payne et al. [1] | Xie et al. [2] | Tamai et al. [3] | Lee et al. [4] |
| --- | --- | --- | --- | --- |
| Year of publication | 2024 | 2024 | 2023 | 2022 |
| Study type | Development study and internal test | Development study and internal test | Development study and internal test | Development study and internal test |
| Study design | Retrospective single-center study | Retrospective single-center study | Retrospective single-center study | Retrospective single-center study |
| Model type | DL ^c^ | DL | DL | DL |
| Primary algorithms used^a^ | ViT ^d^ | ResNet-34 | EfficientNet-B2 | CNN ^j^ |
| Cervical degenerative disease type^b^ | CSM ^e^ | DCS ^h^ | CSM | CSM |
| Data collection period | 2017-2022 | 2021.1-2022.11 | Unmentioned | 2016.6-2021.5 |
| Institution | Stony Brook University, Stony Brook, New York, USA | China Rehabilitation Research Center, Beijing, China | Osaka Metropolitan University Graduate School of Medicine, Osaka, Japan | Yeungnam University Hospital, Daegu, South Korea |
| Number of participants, n | 142 | 595 | 240 | 207 |
| Study population | Patients suspected of CS ^f^ | Patients diagnosed with CS | CSM and controls | Patients suspected of CS |
| Population demographics | Unmentioned | Age: 46.4 ± 16.4 Female: 58.7% | Unmentioned | Age: 58.6±17.5 Female: 42.5% |
| Population source | Emergency, inpatient, and outpatient population | Inpatient and outpatient population | Inpatient population | outpatient population |
| Dataset split details | Training/validation set: 113; Internal test set: 29 | Training/validation set: 1235 ^i^; Internal test set: 136 | Training set: 198; Internal test set: 42 | Training set: 145 ^i^; Internal test set: 62 ^i^ |
| Validation strategy | Hold-out validation | Hold-out validation | Cross-validation and Hold-out validation | Hold-out validation |
| Imaging modality | MRI ^g^ | X-Ray | X-Ray | X-Ray |
| Reference standard | MRI | X-Ray | MRI | MRI |

| Study | Lee et al. [5] | Maraş et al. [6] | Hopkins et al. [7] | Xie et al. [8] |
| --- | --- | --- | --- | --- |
| Year of publication | 2025 | 2022 | 2019 | 2023 |
| Study type | Development study and internal test and external test | Development study and internal test | Development study and internal test | Development study and internal test |
| Study design | Retrospective multi-center study | Retrospective single-center study | Prospective post hoc pilot single-center study | Retrospective multi-center study |
| Model type | DL | DL | DL | TML ^l^ |
| Primary algorithms used^a^ | ResNet-50 and Transformer | VGG-16 | DNN ^k^ | Random Forest |
| Cervical degenerative disease type^b^ | CSM | DCS | CSM | CDDD ^m^ |
| Data collection period | 2013.1-2021.7 | 2019.1-2019.12 | Unmentioned | 2019-2021 |
| Institution | National University Hospital and Ng Teng Fong General Hospital, Singapore | Ankara City Hospital, Ankara, Turkey | Northwestern University Feinberg School of Medicine, Chicago, USA | West China Hospital of Sichuan University, Chengdu, China and First People’s Hospital of Longquanyi District, Chengdu, China |
| Number of participants, n | 504/100 | 331 | 28 | 435 |
| Study population | Patients diagnosed with CS | Patients suspected of CS | CSM and controls | Patients diagnosed with CS |
| Population demographics | Age: 58±13.7 Female: 40% | Unmentioned | Unmentioned | Unmentioned |
| Population source | Inpatient population | rheumatology and immunology outpatient population | recruited participants | Inpatient and outpatient population |
| Dataset split details | Training set: 454; Internal test set: 50; External test set: 100 | Training set: 232; Validation set: 50; Internal test set: 49 | Training set: 18; Internal test set: 10 | Training set: 305; Internal test set: 130 |
| Validation strategy | Hold-out validation | Hold-out validation | Cross-validation | Hold-out validation |
| Imaging modality | MRI | X-Ray | MRI | MRI |
| Reference standard | MRI | X-Ray | MRI | MRI |

| Study | Tachi et al. [9] | Kim et al. [10] | Rhee et al. [11] | Zhang et al. [12] |
| --- | --- | --- | --- | --- |
| Year of publication | 2025 | 2024 | 2025 | 2024 |
| Study type | Development study and internal test | Development study and internal test | Development study and internal test | Development study and internal test |
| Study design | Retrospective single-center study | Retrospective single-center study | Retrospective single-center study | Retrospective single-center study |
| Model type | DL | DL | DL | DL |
| Primary algorithms used^a^ | CNN | DenseNet-161 | VGG-16 | Faster R-CNN |
| Cervical degenerative disease type^b^ | CSM | CSR ^n^ | CSM | CSM |
| Data collection period | 2017.1-2022.12 | 2015.3-2023.11 | 2007.1-2022.12 | 2016.1-2018.12 |
| Institution | Eniwa Hospital, Eniwa City, Japan | Asan Medical Center, University of Ulsan College of Medicine, Seoul, South Korea | Seoul National University Hospital, Seoul, Republic of Korea | Peking University Third Hospital, Beijing, China |
| Number of participants, n | 400 | 1147 | 7645 | 796 |
| Study population | Patients diagnosed with CS | Patients suspected of CS | Patients suspected of CS | Patients diagnosed with CS |
| Population demographics | Unmentioned | Age: 53.4±11.2 Female: 46.1% | Age: 56.1±14.3 Female: 49.4% | Age: 51.1±10.06 Female: 36.4% |
| Population source | Inpatient population | Inpatient and outpatient population | Inpatient and outpatient population | Unmentioned |
| Dataset split details | Training/validation set: 300; Internal test set: 100 | Training set: 798; Validation set: 199; Internal test set: 150 | Training/validation set: 6880; Internal test set: 765 | Training/validation set: 796 |
| Validation strategy | Cross-validation and hold-out validation | Hold-out validation | Hold-out validation | Cross-validation |
| Imaging modality | X-Ray | X-Ray | MRI | MRI |
| Reference standard | MRI | MRI | MRI | MRI |

| Study | Du et al. [13] | Korkmaz et al. [14] | Feng et al. [15] | Zhang et al. [16] |
| --- | --- | --- | --- | --- |
| Year of publication | 2026 | 2024 | 2025 | 2026 |
| Study type | Development study and internal test and external test | Development study and internal test | Development study and internal test and external test | Development study and internal test |
| Study design | Retrospective multi-center study | Retrospective single-center study | Retrospective multi-center study | Retrospective multi-center study |
| Model type | DL | DL | DL | DL |
| Primary algorithms used^a^ | YOLO-11 | CNN | Swin-transformer | ResNet-101 |
| Cervical degenerative disease type^b^ | CSM | CSM | CSM | CSM |
| Data collection period | 2022.3-2024.8 | Unmentioned | 2020.7-2023.6 | 2015.1-2024.10 |
| Institution | Hospital A and Hospital B; specific institution names not reported | Istanbul Kanuni Sultan Suleyman Training and Research Hospital, University of Health Sciences, Istanbul, Turkey | The Fourth People’s Hospital of Shanghai, China, Shanghai Changzheng Hospital, China and Shanghai Changhai Hospital, China | Local hospitals in Wenzhou; specific institution names not reported |
| Number of participants, n | 641/94 | 125 | 795/95 | 425 |
| Study population | Patients suspected of CS | Patients diagnosed with CS | Patients suspected of CS | Patients diagnosed with CS |
| Population demographics | Unmentioned | Age: 56.32±6.77 Female: 46.4% | Age: 55±14 Female: 45.3% | Unmentioned |
| Population source | Unmentioned | Outpatient population | Health checkup and outpatient populations | Unmentioned |
| Dataset split details | Training/validation set: 577; Internal test set: 64; External test set: 94 | Training set: 328 ^i^; Validation set: 163 ^i^ | Training set: 412; validation set: 177; Internal test set: 206; External test set: 95 | Training set (80%); Validation set (10%); Internal test set (10%) |
| Validation strategy | Cross-validation and Hold-out validation | Cross-validation | Hold-out validation | Hold-out validation |
| Imaging modality | MRI | MRI | MRI | CT ^o^ |
| Reference standard | MRI | MRI | MRI | MRI |

| Study | Merali et al. [17] | Yi et al. [18] | Ma et al. [19] | Su et al. [20] |
| --- | --- | --- | --- | --- |
| Year of publication | 2021 | 2023 | 2020 | 2021 |
| Study type | Development study and internal test | Development study and internal test and external test | Development study and internal test | Development study and internal test |
| Study design | Prospective single-center study | Retrospective multi-center study | Retrospective single-center study | Retrospective single-center study |
| Model type | DL | DL | DL | TML |
| Primary algorithms used^a^ | ResNet-50 | ResNet-18 | Faster R-CNN | SVM ^p^ |
| Cervical degenerative disease type^b^ | CSM | CDDD | CDDD and CSM | CSM |
| Data collection period | Unmentioned | 2021,10-2022.1 | 2013.1-2018.12; 2024.5-2024.7 | 2015-2016, 2019-2020 |
| Institution | AO Spine CSM North America and AO Spine CSM International | Beijing Jishuitan Hospital, Beijing, China, Philips Medical Suzhou Co., Ltd, Suzhou, China and The Second Hospital of Zhangjiakou City, Zhangjiakou, China | China-Japan Union Hospital of Jilin University, Jilin, China | Tianjin Medical University General Hospital, Tianjin, China |
| Number of participants, n | 289 | 590/214 | 1500 | 100 |
| Study population | Patients diagnosed with CS | Patients dignosed with CDDD | Patients diagnosed with CS | CSM and controls |
| Population demographics | Unmentioned | Age: 53.9 Female: 36% | Age: Unmentioned Female: 20.7% | Unmentioned |
| Population source | Clinical trial patients | Unmentioned | Inpatient population | Inpatient population and healthy control |
| Dataset split details | Training set: 4941 ^i^; Validation set: 1647 ^i^; External test set: 2991 ^i^ | Validation set: 200 | Training set: 1200; Validation set: 300; Internal test set: 500 ^i^ | Training/validation set: 100 |
| Validation strategy | Hold-out validation | Hold-out validation | Hold-out validation | Cross-validation |
| Imaging modality | MRI | MRI | MRI | MRI |
| Reference standard | MRI | MRI | MRI | MRI |

| Study | Song et al. [21] | Li et al. [22] | Wang et al. [23] | Chen et al. [24] |
| --- | --- | --- | --- | --- |
| Year of publication | 2026 | 2024 | 2025 | 2026 |
| Study type | Development study and internal test and external test | Development study and internal test | Development study and internal test | Development study and internal test and external test |
| Study design | Retrospective multi-center study | Retrospective single-center study | Retrospective single-center study | Retrospective multi-center study |
| Model type | DL | DL | DL | DL |
| Primary algorithms used^a^ | Cascade-Ensemble Deep Learning Framework | Mnasnet | EfficientNet_B5 | ResNet-50 |
| Cervical degenerative disease type^b^ | DCS | DCS | CSM | CSM |
| Data collection period | 2016.1-2018.12 | 2023.7-2024.7 | 2018.1-2021.3 | 2021.1-2025.5 |
| Institution | Peking University Third Hospital, Beijing, China | The Second Affiliated Hospital of Wenzhou Medical University, Wenzhou, China | Fifth Affiliated Hospital of Sun Yat-sen University, Zhuhai, China | Beijing Chaoyang Hospital and Beijing Friendship Hospital, China |
| Number of participants, n | 1151 | 189 | 1000 | 600/60 |
| Study population | Patients diagnosed with CS | Patients diagnosed with CS | Patients diagnosed with CS | Patients suspected of CS |
| Population demographics | Age: 54.0±10.28 Female: 39.4% | Age: 56±15 Female: 41.9% | Unmentioned | Age: 61.10±9.59; Female: 56.7% |
| Population source | Inpatient and outpatient population | Inpatient population | Inpatient and outpatient population | Inpatient population |
| Dataset split details | Training set: 693; Validation set: 228; Internal test set: 230 | Training set: 490 ^i^; Validation set: 146 ^i^; External test set: 120 ^i^ | Training/validation set: 764; Internal test set: 190 | Training set: 480; validation set: 60; internal test set: 60; external test set: 60 |
| Validation strategy | Hold-out validation | Hold-out validation | Hold-out validation | Hold-out validation |
| Imaging modality | X-ray and MRI | MRI | MRI | X-Ray |
| Reference standard | X-ray and MRI | MRI | MRI | MRI |

| Study | Park et al. [25] | Kannan et al. [26] | Arnest et al. [27] |
| --- | --- | --- | --- |
| Year of publication | 2026 | 2025 | 2026 |
| Study type | Development study and internal test and external test | Development study and internal test | Development study and internal test |
| Study design | Retrospective multi-center study | Public dataset-based diagnostic model study | Prospective single-center study |
| Model type | DL | DL | TML |
| Primary algorithms used^a^ | VGG-16 | EfficientNet-B7 | CatBoost |
| Cervical degenerative disease type^b^ | CSM | DCS | CSM |
| Data collection period | 2007.1-2024.12 | Unmentioned | 2019-2025 |
| Institution | Seoul National University Hospital and Bumin Hospital Seoul, Korea | Public datasets: CSXA and F1000 cervical spine dataset | Medical College of Wisconsin, USA; Spine Generic Public Database |
| Number of participants, n | 7878/575 | 258 ^i^ | 130 |
| Study population | Patients suspected of CS | DCS and controls | CSM and controls |
| Population demographics | Age: 53.3±14.0; Female: 50.4% | Unmentioned | Unmentioned |
| Population source | Unmentioned | Public datasets | Unmentioned |
| Dataset split details | Training set: 6302; validation set: 787; internal test set: 789; external test set: 575 | Training set: 756 ^i^; Internal test set: 110 ^i^ | Training set: 80%; Internal test set: 20%; |
| Validation strategy | Hold-out validation | Hold-out validation | Cross-validation and hold-out validation |
| Imaging modality | X-Ray | X-Ray | MRI |
| Reference standard | MRI | MRI | MRI |

| Study | Zhang et al. [28] | Park et al. [29] | Abuhayi et al. [30] |
| --- | --- | --- | --- |
| Year of publication | 2026 | 2023 | 2024 |
| Study type | Development study and internal test and external test | Development study and internal test | Development study and internal test |
| Study design | Retrospective multi-center study | Retrospective single-center study | Retrospective single-center study |
| Model type | DL | DL | DL |
| Primary algorithms used^a^ | PG-nnUNet | ResNet-50 | Inv-AlxVGGNets |
| Cervical degenerative disease type^b^ | DCS | CSR | CDDD |
| Data collection period | Unmentioned | Unmentioned | Unmentioned |
| Institution | Renji Hospital and Ruijin Hospital, Shanghai Jiao Tong University School of Medicine, China | Dongguk University Ilsan Hospital, Korea | University of Gondar Specialized Medical College, Ethiopia |
| Number of participants, n | 960/95 | 798 | 423 |
| Study population | Patients diagnosed with CS | Patients suspected of CS | Patients suspected of CS |
| Population demographics | Unmentioned | Unmentioned | Unmentioned |
| Population source | Unmentioned | Unmentioned | Unmentioned |
| Dataset split details | Training set: 672; Internal test set: 288; External test set: 95 | Training set: 60% i; Validation set: 20% i; Internal test set: 20% i | Training set: 60% i; Validation set: 20% i; Internal test set: 20% i |
| Validation strategy | Hold-out validation | Hold-out validation | Hold-out validation |
| Imaging modality | MRI | X-Ray | MRI |
| Reference standard | MRI | MRI | MRI |

^a^ Algorithms for only classifiers

^b^ Disease types were summarized according to the target condition or imaging task reported in each original study. Broad disease terms were retained when specific imaging findings were not reported separately.

^c^ DL: deep learning

^d^ ViT: vision transformer

^e^ CSM: cervical spondylotic myelopathy

^f^ CS: cervical spondylosis

^g^ MRI: magnetic resonance imaging

^h^ DCS: degenerative cervical spondylosis

^i^ Sample size was based on the number of images

^j^ CNN: convolutional neural network

^k^ DNN: deep neural network

^l^ TML: traditional machine learning. DL as the segmenter and TML as the classifier.

^m^ CDDD: cervical degenerative disc disease

^n^ CSR: cervical spondylotic radiculopathy

^o^ CT: computed tomography

^p^ SVM: support vector machine

Table S2. Assessment of the Independence of Datasets Included in the Meta-Analysis

| Study | Number of datasets | Data Collection Period | Institution | Study Population | Population Source | Population overlap | Independence judgement |
| --- | --- | --- | --- | --- | --- | --- | --- |
| Payne et al. 2024 | 1 | 2017-2022 | Stony Brook University, Stony Brook, New York, USA | Patients suspected of CS | Emergency, inpatient, and outpatient population | No evidence | Independent: distinct institution/data source |
| Xie et al. 2024 (d) | 1 | 2021.1-2022.11 | China Rehabilitation Research Center, Beijing, China | Patients diagnosed with CS | Inpatient and outpatient population | No evidence | Independent: distinct institution/data source |
| Tamai et al. 2023 | 1 | Unmentioned | Osaka Metropolitan University Graduate School of Medicine, Osaka, Japan | CSM and controls | Inpatient population | No evidence | Independent: distinct institution/data source |
| Lee et al. 2022 | 1 | 2016.6-2021.5 | Yeungnam University Hospital, Daegu, South Korea | Patients suspected of CS | outpatient population | No evidence | Independent: distinct institution/data source |
| Lee et al. 2025 | 1 | 2015.11-2020.12 | Ng Teng Fong General Hospital, Singapore | Patients diagnosed with CS | Unmentioned | No evidence | Independent: distinct institution/data source |
| Maras et al. 2022 | 1 | 2019.1-2019.12 | Ankara City Hospital, Ankara, Turkey | Patients suspected of CS | rheumatology and immunology outpatient population | No evidence | Independent: distinct institution/data source |
| Hopkins et al. 2019 | 1 | Unmentioned | Northwestern University Feinberg School of Medicine, Chicago, USA | CSM and controls | recruited participants | No evidence | Independent: distinct institution/data source |
| Xie et al. 2024 (t) | 1 | 2019-2021 | West China Hospital of Sichuan University and First People's Hospital of Longquanyi District, Chengdu, China | Patients diagnosed with CS | Inpatient and outpatient population | No evidence | Independent: distinct institution/data source |
| Tachi et al. 2025 | 1 | 2021.9-2022.12 | Eniwa Hospital, Eniwa City, Japan | Patients diagnosed with CS | Inpatient population | No evidence | Independent: distinct institution/data source |
| Kim et al. 2024 | 1 | 2022.4-2023.11 | Asan Medical Center, University of Ulsan College of Medicine, Seoul, South Korea | Patients suspected of CS | Inpatient and outpatient population | No evidence | Independent: distinct institution/data source |
| Rhee et al. 2025 | 1 | 2007.1-2022.12 | Seoul National University Hospital, Seoul, Republic of Korea | Patients suspected of CS | Inpatient and outpatient population | No evidence | Independent: distinct institution/data source |
| Zhang et al. 2024 | 1 | 2016.1-2018.12 | Peking University Third Hospital, Beijing, China | Patients diagnosed with CS | Unmentioned | No evidence | Independent: distinct institution/data source |
| Du et al. 2026 | 1 | Not separately reported | Hospital B, independent second medical center; specific institution name not reported | Patients suspected of CS | Unmentioned | No evidence | Independent: distinct institution/data source ^a^ |
| Korkmaz et al. 2024 | 1 | Unmentioned | Istanbul Kanuni Sultan Suleyman Training and Research Hospital, University of Health Sciences, Istanbul, Turkey | Patients diagnosed with CS | Outpatient population | No evidence | Independent: distinct institution/data source |
| Feng et al. 2025 | 1 | 2023.6 | Shanghai Changhai Hospital, Shanghai, China | Patients suspected of CS | Health checkup and outpatient populations | No evidence | Independent: distinct institution/data source |
| Zhang et al. 2026 (a) | 1 | 2015.1-2024.10 | Local hospitals in Wenzhou; specific institution names not reported | Patients diagnosed with CS | Unmentioned | No evidence | Independent: distinct institution/data source |
| Chen et al. 2026 | 1 | 2025.1-2025.5 | Beijing Friendship Hospital, Capital Medical University, Beijing, China | Patients suspected of CS | Inpatient population | No evidence | Independent: distinct institution/data source |
| Park et al. 2026 | 1 | 2023.8-2024.12 | Bumin Hospital Seoul, Seoul, Republic of Korea | Patients suspected of CS | Unmentioned | No evidence | Independent: distinct institution/data source |
| Kannan et al. 2025 | 1 | Unmentioned | Public datasets: CSXA and F1000 cervical spine dataset | DCS and controls | Public datasets | No evidence | Independent: distinct institution/data source |
| Arnest et al. 2026 | 1 | 2019-2025 | Medical College of Wisconsin, USA; Spine Generic Public Database | CSM and controls | Unmentioned | No evidence | Independent: distinct institution/data source |
| Zhang et al. 2026 (b) | 1 | Unmentioned | Ruijin Hospital, Shanghai Jiao Tong University School of Medicine, Shanghai, China | Patients diagnosed with CS | Unmentioned | No evidence | Independent: distinct institution/data source |

^a^ No identifiable institutional overlap with other included studies based on reported author affiliations.

Across the 21 studies included in the meta-analysis, no identifiable overlap in institutions, data sources, or study populations was found based on the information reported in the original articles. Each study was therefore treated as contributing one independent dataset to the meta-analysis. However, several studies did not report the data-collection period or detailed population source; consequently, the absence of overlap should be interpreted as “no overlap identified” rather than definitive confirmation of patient-level independence.

Table S3. Assessment of the Independence of External Validation Datasets From Internal Model Development Datasets

| Study | Dataset role | Institution/data source | Data Collection Period | Study Population | Population Source | Independence check |
| --- | --- | --- | --- | --- | --- | --- |
| Lee et al. 2025 | Internal set | National University Hospital, Singapore | 2013.1-2021.7 | Patients diagnosed with CS | Inpatient population | Independent: different institution/data source, although calendar periods overlap |
|  | External set | Ng Teng Fong General Hospital, Singapore | 2015.11-2020.12 | Patients diagnosed with CS | Unmentioned |  |
| Du et al. 2026 | Internal set | Hospital A; specific institution name not reported | 2022.3-2024.8 | Patients suspected of CS | Unmentioned | Independent: different institution/data source ^a^ |
|  | External set | Hospital B, independent second medical center; specific institution name not reported | Not separately reported | Patients suspected of CS | Unmentioned |  |
| Feng et al. 2025 | Internal set | Shanghai Changzheng Hospital | 2021.1-2021.12 | Patients suspected of CS | Health checkup and outpatient populations | Independent: different institution/data source |
|  | External set | Shanghai Changhai Hospital | 2023.6 | Patients suspected of CS | Health checkup and outpatient populations |  |
| Chen et al. 2026 | Internal set | Beijing Chaoyang Hospital, Capital Medical University | 2021.1-2025.5 | Patients suspected of CS | Inpatient population | Independent: different institution/data source, although calendar periods overlap |
|  | External set | Beijing Friendship Hospital, Capital Medical University | 2025.1-2025.5 | Patients suspected of CS | Inpatient population |  |
| Park et al. 2026 | Internal set | Seoul National University Hospital | 2007.1-2022.12 | Patients suspected of CS | Unmentioned | Independent: different institution/data source and non-overlapping periods |
|  | External set | Bumin Hospital Seoul | 2023.8-2024.12 | Patients suspected of CS | Unmentioned |  |
| Zhang et al. 2026 (b) | Internal set | Renji Hospital, Shanghai Jiao Tong University School of Medicine | Unmentioned | Patients diagnosed with CS | Unmentioned | Independent: different institution/data source |
|  | External set | Ruijin Hospital, Shanghai Jiao Tong University School of Medicine | Unmentioned | Patients diagnosed with CS | Unmentioned |  |

^a^ Supported by the authors’ explicit statement that the external dataset originated from an independent second medical center and was not used for model training or hyperparameter tuning.

Du and Park explicitly stated that the external datasets were not used for model training or hyperparameter tuning. Lee explicitly reported that no further training was performed on the external dataset. For Chen, the study author confirmed through direct correspondence that the external dataset was not involved in internal model training or hyperparameter tuning. In Feng and Zhang, the reported dataset partitioning indicated that the external datasets were used for testing or validation. However, the authors did not explicitly state that these datasets were excluded from all model training and hyperparameter-tuning procedures.

1.	Payne DL, Xu X, Faraji F, John K, Pradas KF, Bernard VV, et al. Automated Detection of Cervical Spinal Stenosis and Cord Compression via Vision Transformer and Rules-Based Classification. AJNR American journal of neuroradiology. 2024 Feb 15;45(4):432-8. PMID: 38360785. doi: 10.3174/ajnr.A8141.

2.	Xie Y, Nie Y, Lundgren J, Yang M, Zhang Y, Chen Z. Cervical Spondylosis Diagnosis Based on Convolutional Neural Network with X-ray Images. Sensors (Basel, Switzerland). 2024 May 26;24(11). PMID: 38894217. doi: 10.3390/s24113428.

3.	Tamai K, Terai H, Hoshino M, Tabuchi H, Kato M, Toyoda H, et al. Deep Learning Algorithm for Identifying Cervical Cord Compression Due to Degenerative Canal Stenosis on Radiography. Spine. 2023 Apr 15;48(8):519-25. PMID: 36763843. doi: 10.1097/brs.0000000000004595.

4.	Lee GW, Shin H, Chang MC. Deep learning algorithm to evaluate cervical spondylotic myelopathy using lateral cervical spine radiograph. BMC neurology. 2022 Apr 20;22(1):147. PMID: 35443618. doi: 10.1186/s12883-022-02670-w.

5.	Lee A, Wu J, Liu C, Makmur A, Ting YH, Muhamat Nor FE, et al. Deep learning model for automated diagnosis of degenerative cervical spondylosis and altered spinal cord signal on MRI. The spine journal : official journal of the North American Spine Society. 2025 Feb;25(2):255-64. PMID: 39357744. doi: 10.1016/j.spinee.2024.09.015.

6.	Maraş Y, Tokdemir G, Üreten K, Atalar E, Duran S, Maraş H. Diagnosis of osteoarthritic changes, loss of cervical lordosis, and disc space narrowing on cervical radiographs with deep learning methods. Joint diseases and related surgery. 2022;33(1):93-101. PMID: 35361083. doi: 10.52312/jdrs.2022.445.

7.	Hopkins BS, Weber KA, 2nd, Kesavabhotla K, Paliwal M, Cantrell DR, Smith ZA. Machine Learning for the Prediction of Cervical Spondylotic Myelopathy: A Post Hoc Pilot Study of 28 Participants. World neurosurgery. 2019 Jul;127:e436-e42. PMID: 30922901. doi: 10.1016/j.wneu.2019.03.165.

8.	Xie J, Yang Y, Jiang Z, Zhang K, Zhang X, Lin Y, et al. MRI radiomics-based decision support tool for a personalized classification of cervical disc degeneration: a two-center study. Frontiers in physiology. 2023;14:1281506. PMID: 38235385. doi: 10.3389/fphys.2023.1281506.

9.	Tachi H, Kokabu T, Suzuki H, Ishikawa Y, Yabu A, Yanagihashi Y, et al. Prediction of cervical spondylotic myelopathy from a plain radiograph using deep learning with convolutional neural networks. European spine journal : official publication of the European Spine Society, the European Spinal Deformity Society, and the European Section of the Cervical Spine Research Society. 2025 Sep;34(9):3786-97. PMID: 40381026. doi: 10.1007/s00586-025-08908-8.

10.	Kim J, Yang JJ, Song J, Jo S, Kim Y, Park J, et al. Detection of Cervical Foraminal Stenosis from Oblique Radiograph Using Convolutional Neural Network Algorithm. Yonsei medical journal. 2024 Jul;65(7):389-96. PMID: 38910301. doi: 10.3349/ymj.2023.0091.

11.	Rhee W, Park SC, Kim H, Chang BS, Chang SY. Deep learning-based prediction of cervical canal stenosis from mid-sagittal T2-weighted MRI. Skeletal radiology. 2025 Oct;54(10):2067-76. PMID: 40152984. doi: 10.1007/s00256-025-04917-2.

12.	Zhang E, Yao M, Li Y, Wang Q, Song X, Chen Y, et al. Deep learning model for the automated detection and classification of central canal and neural foraminal stenosis upon cervical spine magnetic resonance imaging. BMC medical imaging. 2024 Nov 26;24(1):320. PMID: 39593012. doi: 10.1186/s12880-024-01489-w.

13.	Du Q, Kong W, Chang Y, Xin Z, Shao X, Feng L, et al. Automated Detection of Cervical Spinal Cord Compression on MRI Using YOLO11 Deep Learning Architecture: A Two-Center External Validation Study. Spine. 2026 2026/05/01;51(9):610-21. PMID: 41631492. doi: 10.1097/BRS.0000000000005639.

14.	Korkmaz M, Yılmaz H, Korkmaz MD, Akgül T. Convolutional Neural Networks in the Diagnosis of Cervical Myelopathy. Revista Brasileira de Ortopedia. 2024 2024/10;59(5):e689-e95. PMID: 39649041. doi: 10.1055/s-0044-1779317.

15.	Feng X, Zhang Y, Lu M, Ma C, Miao X, Yang J, et al. Feasibility of fully automatic assessment of cervical canal stenosis using MRI via deep learning. Quantitative Imaging in Medicine and Surgery. 2025 2025/09/01;15(9):8457-70. PMID: 40893491. doi: 10.21037/qims-2025-67.

16.	Zhang Y-L, Huang J-W, Li K-Y, Li H-L, Lin X-X, Ye H-B, et al. Automated Classification of Cervical Spinal Stenosis Using Deep Learning on Computed Tomography Scans. Spine. 2026 2026/05/15;51(10):717-24. PMID: 40458958. doi: 10.1097/BRS.0000000000005414.

17.	Merali Z, Wang JZ, Badhiwala JH, Witiw CD, Wilson JR, Fehlings MG. A deep learning model for detection of cervical spinal cord compression in MRI scans. Scientific reports. 2021 May 18;11(1):10473. PMID: 34006910. doi: 10.1038/s41598-021-89848-3.

18.	Yi W, Zhao J, Tang W, Yin H, Yu L, Wang Y, et al. Deep learning-based high-accuracy detection for lumbar and cervical degenerative disease on T2-weighted MR images. European spine journal : official publication of the European Spine Society, the European Spinal Deformity Society, and the European Section of the Cervical Spine Research Society. 2023 Nov;32(11):3807-14. PMID: 36943484. doi: 10.1007/s00586-023-07641-4.

19.	Ma S, Huang Y, Che X, Gu R. Faster RCNN-based detection of cervical spinal cord injury and disc degeneration. Journal of applied clinical medical physics. 2020 Sep;21(9):235-43. PMID: 32797664. doi: 10.1002/acm2.13001.

20.	Su Q, Zhao R, Wang S, Tu H, Guo X, Yang F. Identification and Therapeutic Outcome Prediction of Cervical Spondylotic Myelopathy Based on the Functional Connectivity From Resting-State Functional MRI Data: A Preliminary Machine Learning Study. Frontiers in neurology. 2021;12:711880. PMID: 34690912. doi: 10.3389/fneur.2021.711880.

21.	Song X, Li Y, Ouyang H, Yao M, Zhao F, Yu K, et al. Automated diagnostic of cervical spondylosis on multimodal medical images with a multi-task deep learning model. Nature Communications. 2026 2026/02/05;17(1):2392. PMID: 41644944. doi: 10.1038/s41467-026-69023-w.

22.	Li K-Y, Lu Z-Y, Tian Y-H, Liu X-P, Zhang Y-K, Qiu J-W, et al. Deep learning models for MRI-based clinical decision support in cervical spine degenerative diseases. Frontiers in Neuroscience. 2024 2024/12/06;18:1501972. PMID: 39712220. doi: 10.3389/fnins.2024.1501972.

23.	Wang Z, Chen X, Liu B, Hai J, Qiao K, Yuan Z, et al. Deep-Learning-Based Computer-Aided Grading of Cervical Spinal Stenosis from MR Images: Accuracy and Clinical Alignment. Bioengineering. 2025 2025/06/01;12(6):604. PMID: 40564420. doi: 10.3390/bioengineering12060604.

24.	Chen R, Liang M, Zhang Y, Liu X, Wang T, Wang A, et al. Performance comparison between a deep learning model and spine surgeons in detecting cervical spinal cord compression on radiographs. Journal of Neurosurgery: Spine. 2026:1-10. doi: 10.3171/2026.1.spine251409.

25.	Park SC, Rhee W, Chang B-S, Chang SY, Kim H. Development and multi-institutional validation of a deep learning algorithm for predicting cervical cord compression using dynamic cervical lateral radiographs. Scientific reports. 2026. doi: 10.1038/s41598-026-56657-5.

26.	Kannan S, Raman V, Kumar KK, Mohanraj P. CervNet: A Novel Multimodal Neural Network to Diagnose Cervical Spondylosis from X-Ray Images. International Journal of Advanced Science and Engineering. 2025;12(1):4871-84. doi: 10.29294/ijase.12.1.2025.4871-4884.

27.	Arnest RM, Koch KM, Budde MD, Banerjee A, Vedantam A. Machine learning-based MRI radiomics identifies patients with degenerative cervical myelopathy and predicts baseline function. European Spine Journal. 2026. doi: 10.1007/s00586-026-10043-x.

28.	Zhang Q, Chen X, He Z, Wu L, Wang K, Sun J, et al. Pathology-Guided AI System for Accurate Segmentation and Diagnosis of Cervical Spondylosis. IEEE journal of biomedical and health informatics. 2026;30(2):1216-29. doi: 10.1109/jbhi.2025.3598469.

29.	Park J, Yang J, Park S, Kim J. Deep Learning-Based Approaches for Classifying Foraminal Stenosis Using Cervical Spine Radiographs. Electronics. 2023;12(1):195. doi: 10.3390/electronics12010195.

30.	Abuhayi BM, Agegnehu Bezabh Y, Melese Ayalew A. Inv-AlxVGGNets: Cervical Spine Disease Classification Using Concatenated Involutional Neural Networks With Residual Net. IEEE Access. 2024;12:102188-201. doi: 10.1109/access.2024.3432803.
